# A systematic review and meta‐analysis of studies that have evaluated the role of mitochondrial function and iron metabolism in frailty

**DOI:** 10.1111/cts.13101

**Published:** 2021-07-09

**Authors:** Kristina Tomkova, Suraj Pathak, Riccardo Abbasciano, Marcin Wozniak, Gavin J. Murphy

**Affiliations:** ^1^ Department of Cardiovascular Sciences and National Institute for Health Research Leicester Biomedical Research Unit in Cardiovascular Medicine University of Leicester Leicester UK

## Abstract

Frailty is a condition of global impairment due to depletion of physiological reserves. However, the underlying biological mechanisms are poorly understood. The aims of the current study were to identify the differences in mitochondrial function and iron metabolism between frail and nonfrail populations, and to investigate the contribution of different methodological approaches to the results. Searches were performed, using five online databases up to November 2019. Studies reporting measurements of mitochondrial function or iron metabolism in frail and nonfrail subjects or subjects with and without sarcopenia, were included. Pooled effect estimates were expressed as Standardized Mean Differences. Heterogeneity, expressed as *I* ^2^, was explored using regression analyses. In total, 107 studies, reporting 75 measures of mitochondrial function or iron metabolism, using six different experimental approaches, in three species were identified. Significant decreases in measures of oxygen consumption were observed for frail humans but not in animal models. Conversely, no differences between frail and nonfrail humans were observed for apoptosis and autophagy, in contrast to animal models. The most significant effect of the type of frailty assessment was observed for respiratory chain complexes where only subjects categorized as frail by the Fried Frailty Index showed a significant decrease in activity. We identified iron metabolism in frailty as an important knowledge gap, highlighted the need of consistent frailty diagnostic tools, and pointed out the limited translational potential of animal models. Inconsistency between studies evaluating the molecular mechanisms underlying frailty may present a barrier to the development of effective therapies.

**Study Highlights**

**WHAT IS THE CURRENT KNOWLEDGE ON THE TOPIC?**

The phenotype of frailty has been described in depth in current literature. However, the molecular mechanism underlining this syndrome remain unclear.

**WHAT QUESTION DID THIS STUDY ADDRESS?**

In order to clarify the role of mitochondrial function and iron metabolism in frailty, we systematically reviewed and meta‐analyzed all available data examining these processes in frail versus non‐frail subjects.

**WHAT DOES THIS STUDY ADD TO OUR KNOWLEDGE?**

This review identified mitochondrial dynamics, oxygen consumption, and the activities and abundancies of mitochondrial respiratory complexes as main categories of mitochondrial function dysregulated in frailty, and we believe these provide a core dataset of outcomes for future research in this area.

**HOW MIGHT THIS CHANGE CLINICAL PHARMACOLOGY OR TRANSLATIONAL SCIENCE?**

This study also identified a significant difference between the dysregulated molecular pathways in animal models and human subjects, pointing out the minuscule translational potential of frailty research using current animal models of frailty. Furthermore, our findings strongly underline the need for more consistent frailty definition, as the heterogeneity of methodological approaches and frailty assessment tools were a barrier to gaining consistent and easily interpretable results. We believe that the findings and recommendations presented in this review provide novel insights into molecular mechanism of frailty and present a valuable resource for future translational studies.

## INTRODUCTION

Frailty is a clinical syndrome characterized by loss of cognitive function, sarcopenia, unintentional weight loss, and low energy. In clinical terms, this translates into increased vulnerability to otherwise minor stressors, that can lead to prolonged hospitalization, or the requirement for social care or even long‐term convalescent care.^1^ Frailty is chiefly associated with advancing age but can occur in younger people with additional risk factors, including sedentary lifestyle, poor nutrition, chronic disease, and chronic inflammatory states. As the population ages, and as the numbers of patients with multiple chronic conditions, or cardiometabolic disease, increases, frailty will present an increasing challenge to clinicians and health systems. The frailty phenotype is poorly defined, with over 25 frailty definitions in current clinical use.^2^ This reflects the limited understanding of the underlying mechanisms. Existing research points toward a multifactorial pathophysiology characterized by loss of mitochondrial function in skeletal muscle, altered iron metabolism, and exposure to oxidative stress.^3^, ^4^ The primary aim of the current study was to summarize and critically review all published studies that have measured these processes in experimental or human studies of frailty. A secondary aim was to evaluate the strengths and limitations of different measures of mitochondrial function or iron metabolism with a view to application in future translational studies of the frailty phenotype.

## MATERIALS AND METHODS

A systematic review of randomized controlled trials and animal studies was performed using the methods described in the Cochrane Handbook for Systematic Reviews of Interventions.^5^ The study adhered to the Preferring Reporting Items for Systematic Reviews and Meta‐Analyses (PRISMA) guidelines.^6^

### Study eligibility

We included studies reporting measurements of iron metabolism or mitochondrial function in frail and nonfrail subjects or subjects with and without sarcopenia, irrespective of blinding, date of publication, sample size, or race of subjects. Review studies, studies examining nonmammalian populations, and studies examining juvenile populations were excluded.

### Information sources

PubMed, Cochrane library, Ovid Medline, Scopus, and BioRXiv databases were searched using variations of search terms: (frail* OR “frailty syndrome” OR “frail elderly” OR sarcopenia) AND (iron OR dmt1 OR ferroportin OR transferrin OR ferritin OR hepcidin OR “circulating iron” OR anemia OR mitochondri* OR gdf15 OR vimentin OR ldh). The final search was performed on November 25, 2019. A full description of the search terms is listed in the Table S1.

### Study selection

Studies identified in online searches were managed using Endnote X9. Title, abstract, and full text screening were carried out and excluded studies and the reason for exclusion were recorded.

### Assessment of methodological quality and publication bias

The methodological quality and the risk of bias for human studies were assessed using a modified version of the Newcastle ‐ Ottawa Quality Assessment Scale for Human Cohort Studies (Supplementary File S1).^7^ For animal studies, the Animal Research: Reporting In Vivo Experiments (ARRIVE) checklist was used, as described previously.^8^

### Outcomes of interest

The prespecified coprimary outcomes or this review were measures of mitochondrial function and iron metabolism.

### Data extraction

Data extraction was completed using a standardized pro forma as follows: authors, publication date, journal name, study title, research aim, main findings, species, and the method used for assessment of frailty. For each measured variable in a given study, the mean and the SD were extracted as well as the number of subjects in the frail and nonfrail groups. To extract the numerical data presented only graphically, the WebPlot Digitizer 4.2 software was utilized.^9^ For studies assuming frailty based on age of the human/animal model, the oldest group was considered frail and the youngest adult group was considered the control/nonfrail group. Where human subjects/animal models were assessed as prefrail, these were classified as frail in the final analysis. Where a single study measured biomarkers of frailty in multiple tissue types, only measurements from one type of tissue was included, based on the most common tissue type assessed across all studies investigating this outcome. Studies reporting only median were excluded from the analysis, as the mean and SD could not be estimated from the reported data.

### Data synthesis and measures of effect sizes

Standardized mean difference (SMD) with (95% confidence intervals [CIs]) and *p* values for effect sizes were estimated for continuous outcomes. The analysis was performed using R programming software with the “*metafor*” package.^10^, ^11^ Because the included studies likely do not share the same effect size, random effects models were fitted using the rma function with the restricted maximum‐likelihood (REML) estimator.^12^

### Dealing with heterogeneity

Prespecified sources of heterogeneity included species and methods of frailty or sarcopenia assessment. The Cochran *Q* test was used to test for heterogeneity between studies in random models.^13^ The *I* ^2^ statistic was used to estimate the percentage of total variation across studies attributed to heterogeneity, rather than chance. Heterogeneity was defined as:

- *I* ^2^ = 0%–40%: no or mild heterogeneity
- *I* ^2^ = 40%–80%: moderate heterogeneity
- *I* ^2^ > 80%: severe heterogeneity

For all variables, random models with prespecified sources of heterogeneity as moderators were fitted using the rma function of the *metafor* package.^11^ For clarity, pooled effect estimates were reported for all prespecified subgroups.

## RESULTS

### Characteristics of included studies

**FIGURE 1:**
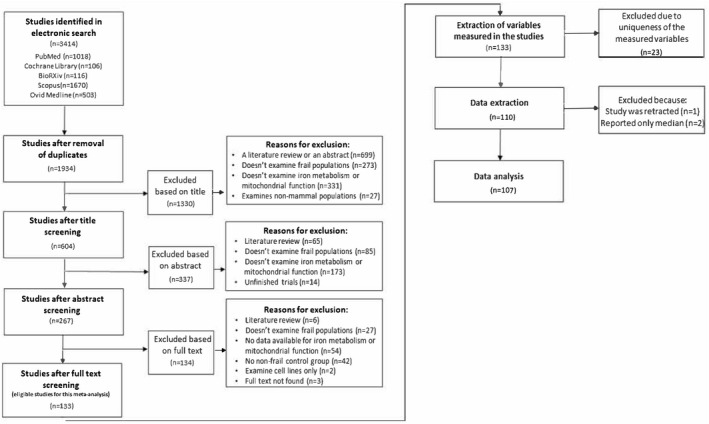
Preferring Reporting Items for Systematic Reviews and Meta‐Analyses (PRISMA) flow chart of the selection of studies for this review

A flow diagram showing the results of the searches and exclusions is shown in Figure 1. In total, 107 studies were included in the data synthesis (Supplementary File S2). The included studies evaluated a total of 75 different measures of mitochondrial function across three species: human (*n* = 32), rat (*n* = 34), and mouse (*n* = 41). Frailty was assessed using six different approaches: age (*n* = 57), age and immobilization/ sedentary lifestyle (*n* = 13), sarcopenia (*n* = 8), Fried Frailty Index (*n* = 10), other geriatric assessments (*n* = 4), or genetically modified animal models of frailty (*n* = 15). The main findings of individual studies and their detailed characteristics are reported in the supplementary files (Supplementary File S3). The full list of assessed variables, including the frequencies of the prespecified sources of heterogeneity and other characteristics, is presented in the Supplementary File S4.

### Assessment of methodological quality

**FIGURE 2:**
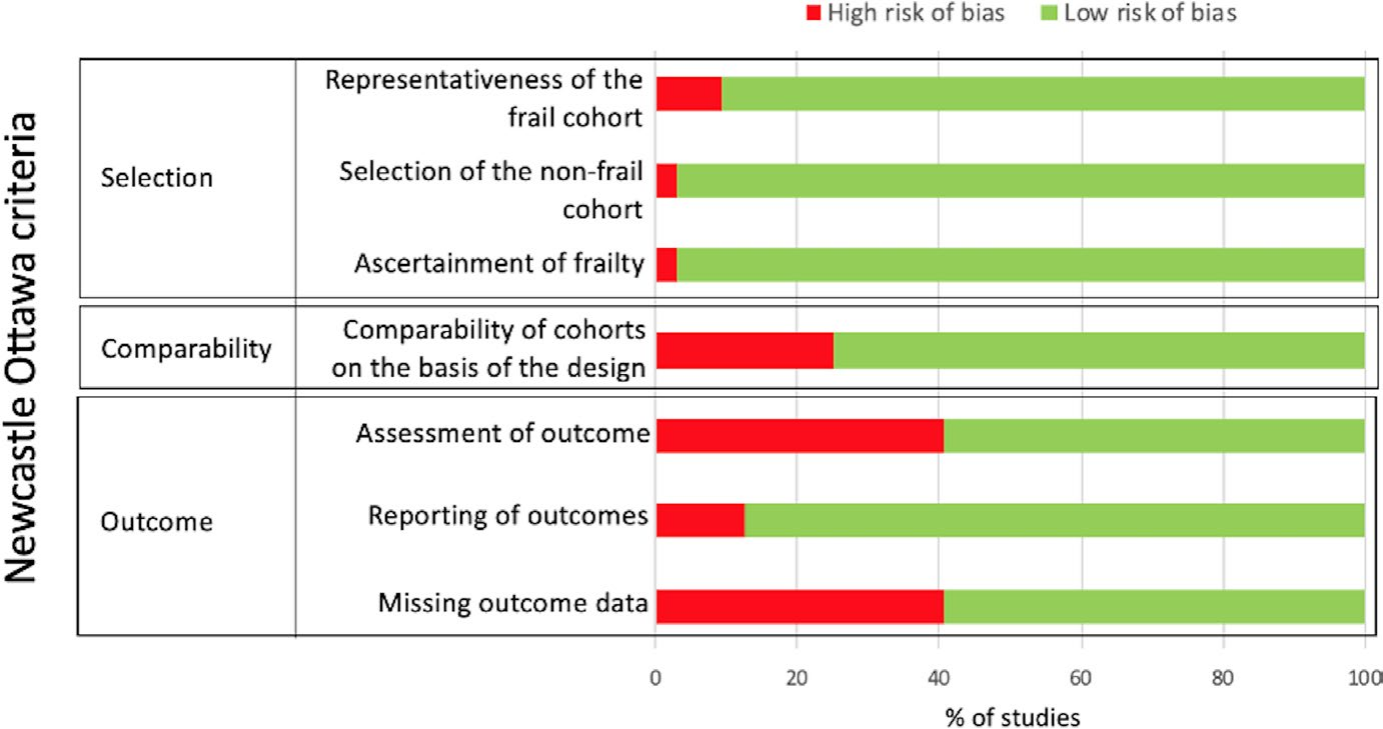
The results of the assessment of the methodological quality in human studies

Using the Newcastle Ottawa scale for studies including humans, three out of 32 were found to be without methodological limitations (Figure 2). For the other human studies, the most common limitations were missing records of blinding and randomization (12/32, 41%) and more than 20% of participants having missing data (12/32, 41%).

**FIGURE 3:**
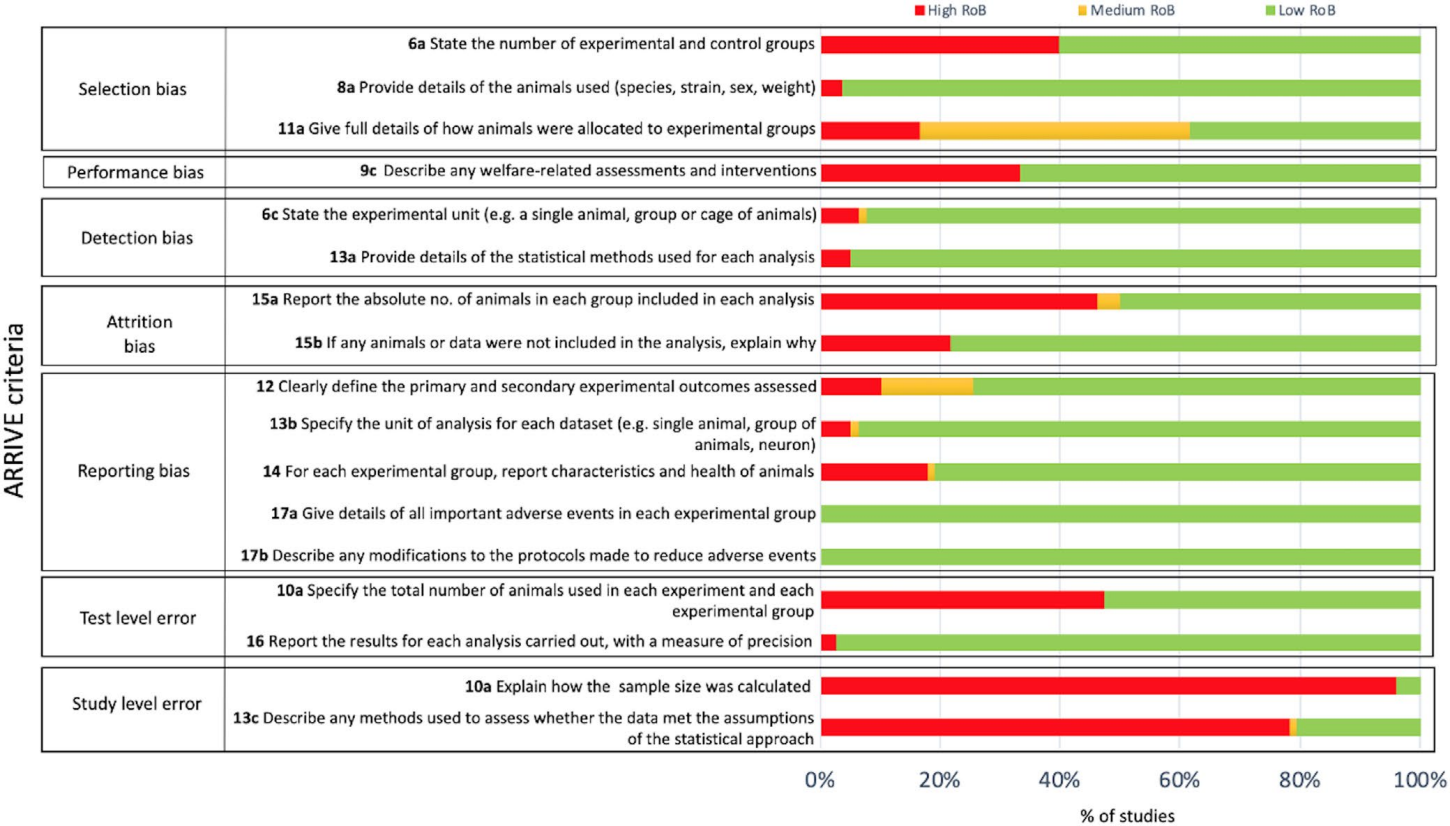
The results of the assessment of the methodological quality in animal studies

Using the ARRIVE checklist for studies, including experimental animals, no study was found to fulfil all quality criteria; therefore, all studies were considered at high risk of bias (Figure 3). The most common methodological limitations included failure to provide a sample size calculation (76/78, 96%), missing description of methods used to prove that the analyzed data met the assumptions of the statistical approach used in the study (63/78, 78%), failure to report the exact numbers of subjects included in each group in each analysis (36/78, 46%), and failure to report the absolute number of animals studied in each experiment (37/78, 47%).

### Data synthesis

**FIGURE 4:**
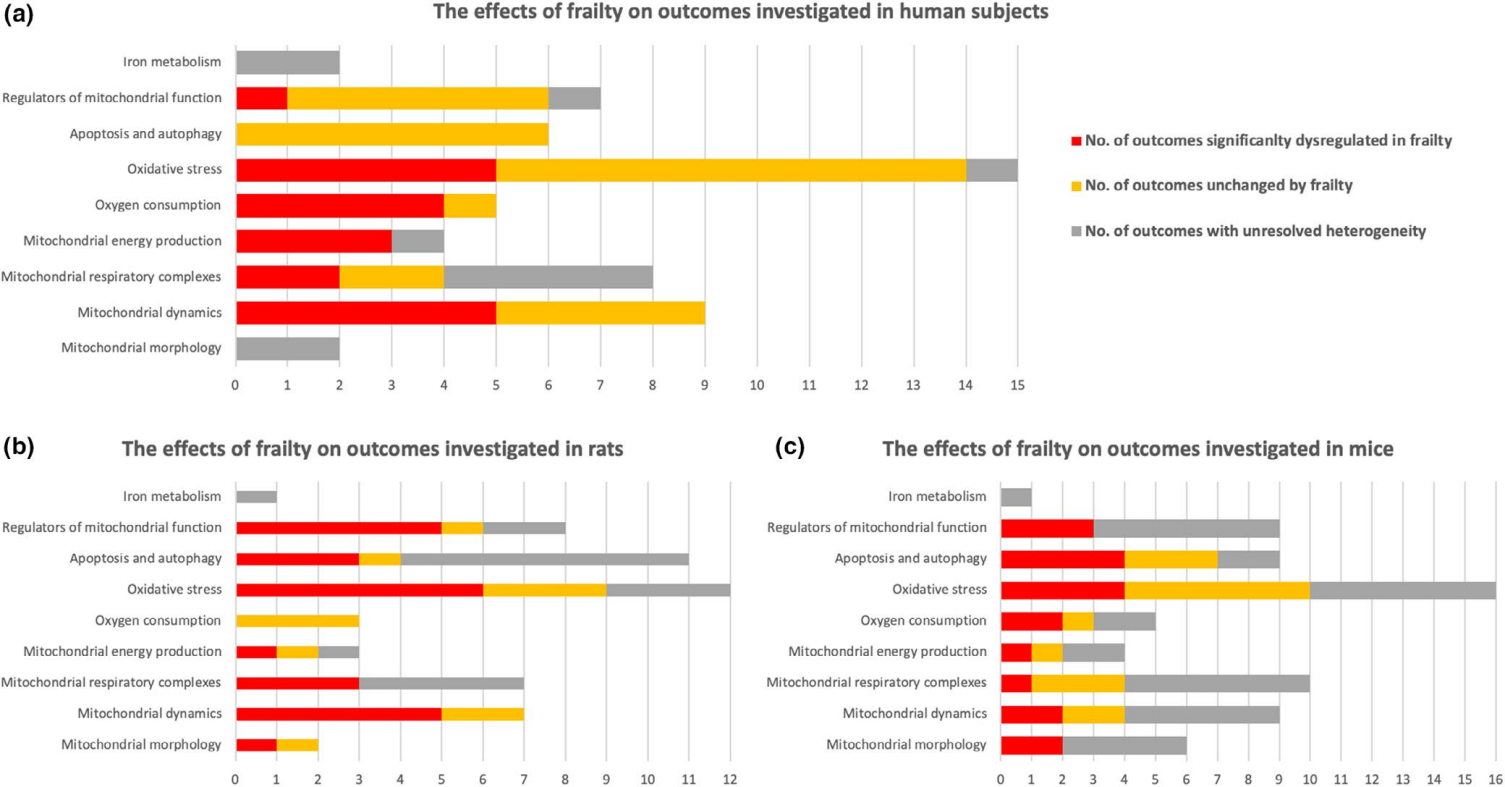
This figure shows the effects of frailty on measures of mitochondrial function and iron metabolism in (a) humans, (b) rats, and (c) mice. The measures of mitochondrial function and iron metabolism are divided into nine functional categories: (1) iron metabolism, (2) regulators of mitochondrial function, (3) apoptosis and autophagy, (4) oxidative stress, (5) oxygen consumption, (6) mitochondrial energy production, (7) mitochondrial complexes, (8) mitochondrial dynamics, and (9) mitochondrial morphology. The horizontal axis represents the number of outcomes examined in the given category. In each category, the proportions of outcomes are highlighted which are dysregulated (red), unaffected (yellow), and unresolved (grey) in frailty

Multiple measures of frailty and iron metabolism were identified, and these were further categorized into the subheadings described below. The effects of frailty on the outcomes in these categories for humans, mice, and rats are shown in Figure 4.

### Mitochondrial morphology

Estimates of mitochondrial number, mitochondrial size, and mitochondrial (mt) DNA copy number in frail and nonfrail humans and animals showed severe heterogeneity between studies that was not resolved by moderator and subgroup analyses (Supplementary File S5, Table S2). Mitochondrial volume density and voltage‐dependent anion channel (VDAC) protein expression did not differ significantly between the frail and nonfrail groups, without heterogeneity. Studies measuring mtDNA/nuclear DNA ratio reported significantly lower values in the frail compared to the nonfrail groups, without heterogeneity (Supplementary File S5, Table S2).

### Mitochondrial dynamics

The expression of genes and proteins that regulate mitochondrial dynamics showed differential regulation within individual subgroups. The gene expression of Dynamin related protein (DRP) 1, a regulator of mitochondrial fission, was downregulated in frail humans and rats but not in genetically modified mice (Supplementary File S5, Table S3). In contrast, protein expression of DRP 1 and Mitochondrial fission 1 protein (FIS1) were increased in rats and mice, respectively, but not in humans.

Expression of mitochondrial fusion genes Mitofusin (MFN) 1, MFN 2, and Mitochondrial Dynamin Like GTPase (OPA1), were reduced in frail groups across species, although there was residual heterogeneity of the estimates for these genes in studies where frailty was defined by age. Conversely, protein expression of MFN1 was not different between frail and nonfrail humans and animals across all moderators and subgroups. MFN2 protein expression was increased in experimental models where frailty was defined by age, but not in other subgroups. OPA1 protein expression was reduced in human models of frailty, but not in other species, and the effects were inconsistent across different frailty definitions.

### Mitochondrial respiratory complexes

Heterogeneity for gene and protein expression and activity of the oxidative phosphorylation complexes were explained by the frailty definition used (Supplementary File S5, Table S4). When the Fried Frailty Index was used in human studies, all outcomes (complex I–V activity, gene and protein expression), with the exception of activity of respiratory complex I, were significantly reduced in subjects classified as frail. For genetically modified animal models, only protein and gene expression of the respiratory complexes were reduced, whereas the enzymatic activities of these complexes, with the exception of complex I, were unaffected. When age or immobilization/sedentary lifestyle were used as measures of frailty, the heterogeneity for the majority of the outcomes was severe, and where the heterogeneity did not reach significant levels, there was no difference between the frail and nonfrail groups. Only a small number of outcomes in this category was examined in studies using geriatric assessment tools or sarcopenia as a measure of frailty, limiting the assessment of these studies.

### Mitochondrial energy production

Severe heterogeneity for comparisons of 31P‐MRS phospho‐creatine recovery rate, citrate synthase protein expression, and citrate synthase activity, between frail and nonfrail could not be resolved using either species or type of frailty assessment as moderators (Supplementary File S5, Table S5). ATP levels were significantly lower in frail rats, but for mice the residual heterogeneity remained severe. Phosphate/oxygen (P/O) ratio, defined as the number of atoms of phosphorus incorporated into an ATP molecule per every two electrons used for reduction of the O_2_ molecule in OXPHOS, was significantly decreased in frail humans and mice, however, only three studies contributed to this estimate.

### Oxygen consumption

Frailty was associated with significantly lower oxygen consumption in both state 3 and state 4, as well as maximal oxygen uptake (VO2_max_) in humans, without heterogeneity (Supplementary File S5, Table S6). In contrast, there was no difference in oxygen consumption measured in frail versus nonfrail rats. Mouse studies demonstrated high heterogeneity for these outcomes, and even for outcomes where the heterogeneity was resolved, no consistent influence of frailty on oxygen consumption was seen.

### Oxidative stress

Assessments of the role of oxidative stress in frailty demonstrated discordant observations between species. In human studies, there were no significant differences between frail and nonfrail subjects with respect to reactive oxygen species (ROS) production or protein carbonylation (Supplementary File S5, Table S7). Antioxidant enzyme levels were unchanged or lower in frail subjects in humans. In contrast, in mouse studies, the production of ROS in respiratory state 2 and mitochondrial ROS production were increased in frail animals and the levels of catalase and SOD1 proteins as well as the activity of GPX were higher (Supplementary File S5, Table S7). The small numbers of studies for many of the examined outcomes limited the certainty of these results. For rat studies, an increase in ROS production was seen in states 3 and 4 respiration but not for state 2, or for mitochondrial ROS production. The effects of frailty on antioxidant proteins in rats could not be examined due to the high heterogeneity and the small number of reported outcomes.

### Autophagy and apoptosis

Measures of apoptosis and autophagy in frailty differed significantly between species. In human studies, there were no differences between frail and nonfrail subjects for any measures of autophagy or apoptosis (Supplementary File S5, Table S8). In rat studies, the apoptotic markers apoptosis inducing factor (AIF) release, and caspase 3 activity, were increased, and the autophagy markers microtubule‐associated protein 1A/1B‐light chain 3 (LC3) and Beclin1 protein expressions were reduced. There was severe heterogeneity for other reported measures in rat studies. In mouse studies, no consistent differences in markers of apoptosis or autophagy were seen, although the numbers of included studies reporting these outcomes was small.

### Regulators of mitochondrial function

In human studies, out of the examined outcomes, only Sirtuin 3 (SIRT3) protein expression was shown to differ between frail and nonfrail subjects (Supplementary File S5, Table S9). In rats, peroxisome proliferator‐activated receptor gamma coactivator 1‐alpha (PGC1α) gene expression, insulin‐like growth factor 1 (IGF1) levels, nuclear respiratory factor 1 (NRF1), and SIRT3 protein expressions were lower in frail animals. However, NRF1 gene expression was unchanged and the mitochondrial transcription factor A (TFAM) protein expression was higher in frail rats. In mouse studies, the severe heterogeneity for all of the reported outcomes limited analyses.

### Measures of iron metabolism

Only three measures of iron metabolism were identified in the studies included in this review. Hemoglobin concentrations in blood were significantly lower in frail human and animal subjects across all subgroups, with the exception of one study examining human subjects with sarcopenia/muscle atrophy (Supplementary File S5, Table S10).

Non‐heme iron levels in tissue were significantly increased in frail human and animal subjects across all subgroups. There was severe heterogeneity for serum transferrin levels between studies that was not resolved by subgroup analysis.

## DISCUSSION

There were three major finding of the current analysis:

First, there is significant heterogeneity for the reported effects of frailty on individual outcomes between species. For example, measures of oxidative stress as well as measures of autophagy and apoptosis were not different between frail and nonfrail humans, but there was evidence of changes in these processes in frail versus nonfrail rats and mice. The examination of mitochondrial dynamics markers provided the most consistent data across species, where the observed outcomes showed similar patterns of dysregulation for both humans and rats. However, even in this category, the frail mice differed significantly from frail rats and humans. This trend was consistent for the majority of the outcomes reported in this review, suggesting that there is very little homology between frailty in mice and humans. We believe that this discordance arises from the difficulty to mimic this complex syndrome with simple genetic modifications, or aging, which were the two most common models of frailty used in mouse studies.

Second, the frailty definitions utilized for characterization of frail groups have significant effects on the results. For example, frailty is associated with reduction in respiratory complex activity, gene expression, and protein expression in humans. However, in other subgroups, this dysregulation is not visible. Moreover, our analyses showed that regardless of species, in subgroups where the definition of frailty allowed for more subjective categorization of examined subjects (based on age and sedentary lifestyle), the heterogeneity for majority of outcomes was severe and could not be resolved. These findings strongly highlight the need for more consistent frailty definitions and suggest that the processes dysregulated in frailty are distinct from aging.

Third, the inconsistent experimental design, data definitions, and reporting resulted in severe heterogeneity across multiple analyses and is likely to present a barrier to clinical progress in this important area of research. The severe heterogeneity observed for almost all outcomes limits our ability to identify a set of core measures that might be put forward for consistency of reporting. However, when considering only human studies, the heterogeneity for most outcomes was nonsignificant and three categories were identified as consistently dysregulated in frailty: mitochondrial dynamics, oxygen consumption, and the activities and abundancies of respiratory complexes. We believe a semitargeted examination of these aspects of mitochondrial function is a reasonable core dataset for future research in this area.

A final comment is that our study failed to identify detailed investigations of dysregulated iron metabolism, despite clear differences in heme and tissue iron levels in experimental models. We suggest that this is a knowledge gap that requires further study.

### Strengths and limitations

The study has three important strengths. First, to our knowledge, this is the most comprehensive analysis of the role of mitochondrial dysfunction in the frailty phenotype to have been performed. Second, we used comprehensive searches, contemporary assessments of methodological quality, and statistical methods to quantify the contribution of prespecified subgroups to heterogeneity observed in the analyses. Third, the broad eligibility criteria provided a unique perspective on the available evidence, across human and nonhuman studies that have addressed this research question. Together, these strengths identified, for the first time, important distinctions between molecular markers of frailty in animal models, and in humans, the limitations of different frailty phenotypes that may reflect measures of convenience; age, rather than more precise definitions of frailty, and will inform a more considered discussion of how reporting of these analyses might be improved.

The main limitation of the study is the heterogeneity observed across almost all of the analyses. We anticipated heterogeneity in the analyses using standardized mean differences for measurements of continuous outcomes, and random effects for the primary meta‐analysis. We also prespecified two likely sources of heterogeneity, however, in many cases, adjustment for these factors did not resolve inconsistent results. The assessments of methodological quality identified many potential limitations, and these may have contributed to the results. Variability in the quality of reporting also raises concerns that other unreported factors contributing to heterogeneity were not controlled for. It is also important to consider that outcome measures are often chosen for pragmatism, cost, and the requirement to answer specific hypotheses. Overall, we consider that the most plausible explanation for the observed heterogeneity was indeed attributable to differences in experimental approach. Standardized reporting of outcomes in this area of research, the wider use of untargeted techniques, and the use of direct measurement of mitochondrial function, rather than indirect measurement, may lead to more consistent and easily interpretable results.

## CONCLUSIONS AND SUGGESTIONS FOR FUTURE WORK

In conclusion, our findings show that frailty is characterized by dysregulated mitochondrial function; mainly changes in oxygen consumption, mitochondrial dynamics, and activities and abundancies of the mitochondrial respiratory complexes. Conversely, the frailty in animal models was characterized by increased oxidative stress, apoptosis, and autophagy. Therefore, for future studies in this area we strongly recommend a shift in focus to examination of human subjects as opposed to research in animal models. Only two studies examined non‐rodent animal models (dogs^14^ and monkeys^15^), resulting in lack of evidence to either confirm or deny their translational potential in frailty research. We therefore further recommend a more extensive evaluation of larger species as animal models for the frailty syndrome, which could prove to be an improvement to the current rodent animal model.

The high heterogeneity observed across this study significantly limited detailed characterization of the molecular processes underlying these changes. These findings highlight the need for comprehensive rules for frailty diagnosis, experimental design, and reporting. Therefore, for future studies we further emphasize the importance of the use of comprehensive and validated frailty scales and indexes as an alternative the use of more subjective categorization tools based on the age of the subjects.

This study also identified a significant knowledge gap, as only three measures of iron metabolism were identified in this work. Therefore, we believe the role of iron metabolism in frailty should be prioritized in future research order to advance this field.

## CONFLICT OF INTERESTS

Professor Murphy declares a financial relationship with Zimmer Biomet and Terumo, outside the scope of the current work. All other authors declared no competing interests for this work.

## AUTHOR CONTRIBUTIONS

K. T. and G. J. M. wrote the manuscript; M. W. designed the research; K. T., S. P. and R. A. performed the research; K. T. analyzed the data.

## Supporting information

File S1

File S2

File S3

File S4

File S5

Table S1
