# Supplementary material for: A systematic review and meta‐analysis of studies that have evaluated the role of mitochondrial function and iron metabolism in frailty
Source: Clin Transl Sci. 2021 Jul 9;14(6):2370–8. doi: 10.1111/cts.13101 (PMC8604243; doi:10.1111/cts.13101)
Supplement: Supplementary file 1 — File S1 [file CTS-14-2370-s002.doc]

**NEWCASTLE - OTTAWA QUALITY ASSESSMENT SCALE**

**COHORT STUDIES**

A study can be awarded a maximum of one star for each numbered item within the Selection and Outcome categories. A maximum of two stars can be given for Comparability.

Overall maximum points is 8 for any individual study.

**Selection**

1) **Representativeness of the frail cohort**

a) truly representative of the average frail community **(1 point)**

b) somewhat representative of the frail community **(1 point)**

c) selected specific group

d) no description of the derivation of the cohort

2) **Selection of the non-frail cohort**

a) drawn from the same community as the frail cohort **(1 point)**

b) drawn from a different source

c) no description of the derivation of the non-frail cohort

3) **Ascertainment of frailty**

a) secure record (e.g. physical testing) **(1 point)**

b) structured interview **(1 point)**

c) written self-report

d) no description

**Comparability**

1) **Comparability of cohorts on the basis of the design or analysis**

a) study controls for main confounding factors **(1 point)**

b) study controls for any additional factor **(1 point)**

**Outcome**

1) **Assessment of outcomes**

a) independent blind assessment **(1 point)**

b) record linkage **(1 point)**

c) self-report

d) no description

2) **Reporting of outcomes**

a) followed pre-specified plan **(1 point)**

b) reported selected outcomes only

d) no description

3) **Missing outcome data**

a) the outcome is measured in all participants **(1 point)**

b) the proportion of missing outcome data is sufficiently low **(1 point)**

c) large proportion of missing data

d) no description
