# Supplementary material for: A systematic review and meta‐analysis of studies that have evaluated the role of mitochondrial function and iron metabolism in frailty
Source: Clin Transl Sci. 2021 Jul 9;14(6):2370–8. doi: 10.1111/cts.13101 (PMC8604243; doi:10.1111/cts.13101)
Supplement: Supplementary file 2 — File S2 [file CTS-14-2370-s004.docx]

**Studies included in our work:**

1. Achin, N.A., et al., *Behavioral assessment and blood oxidative status of aging sprague dawley rats through a longitudinal analysis.* Current Aging Science, 2018. **11**(3): p. 182-194.
2. Altun, M., et al., *Iron load and redox stress in skeletal muscle of aged rats.* Muscle & Nerve, 2007. **36**(2): p. 223-33.
3. Andreux, P.A., et al., *Mitochondrial function is impaired in the skeletal muscle of pre-frail elderly.* Scientific Reports, 2018. **8**(1): p. 8548.
4. Arai, Y., et al., *Lipoprotein metabolism in Japanese centenarians: effects of apolipoprotein E polymorphism and nutritional status.* Journal of the American Geriatrics Society, 2001. **49**(11): p. 1434-41.
5. Arinzon, Z., et al., *Functional recovery after hip fracture in old-old elderly patients.* Archives of gerontology and geriatrics, 2005. **40**(3): p. 327‐336.
6. Ascenzi, F., et al., *Effects of IGF-1 isoforms on muscle growth and sarcopenia.* Aging Cell, 2019. **18**(3): p. e12954.
7. Bahreinipour, M.A., et al., *Mild aerobic training with blood flow restriction increases the hypertrophy index and MuSK in both slow and fast muscles of old rats: Role of PGC-1α.* Life Sciences, 2018. **202**: p. 103-109.
8. Barani, A.E., et al., *Age-related changes in the mitotic and metabolic characteristics of muscle-derived cells.* Journal of Applied Physiology, 2003. **95**(5): p. 2089-98.
9. Beltran Valls, M.R., et al., *Protein carbonylation and heat shock proteins in human skeletal muscle: relationships to age and sarcopenia.* Journals of Gerontology Series A-Biological Sciences & Medical Sciences, 2015. **70**(2): p. 174-81.
10. Betik, A.C., et al., *Exercise training from late middle age until senescence does not attenuate the declines in skeletal muscle aerobic function.* American Journal of Physiology - Regulatory Integrative and Comparative Physiology, 2009. **297**(3): p. R744-R755.
11. Bourdel-Marchasson, I., et al., *Muscle phosphocreatine post-exercise recovery rate is related to functional evaluation in hospitalized and community-living older people.* Journal of Nutrition, Health & Aging, 2007. **11**(3): p. 215-21.
12. Brioche, T., et al., *Growth hormone replacement therapy prevents sarcopenia by a dual mechanism: improvement of protein balance and of antioxidant defenses.* Journals of Gerontology Series A-Biological Sciences & Medical Sciences, 2014. **69**(10): p. 1186-98.
13. Bunout, D., et al., *Relationship between protein and mitochondrial DNA oxidative injury and telomere length and muscle loss in healthy elderly subjects.* Archives of Gerontology and Geriatrics, 2009. **48**(3): p. 335-339.
14. Campbell, M.D., et al., *Improving mitochondrial function with SS-31 reverses age-related redox stress and improves exercise tolerance in aged mice.* Free Radical Biology & Medicine, 2019. **134**: p. 268-281.
15. Capel, F., et al., *Differential variation of mitochondrial H2O2 release during aging in oxidative and glycolytic muscles in rats.* Mechanisms of Ageing and Development, 2004. **125**(5): p. 367-373.
16. Chabi, B., et al., *Mitochondrial function and apoptotic susceptibility in aging skeletal muscle.* Aging Cell, 2008. **7**(1): p. 2-12.
17. Chen, L.H., et al., *Lactobacillus paracasei PS23 decelerated age-related muscle loss by ensuring mitochondrial function in SAMP8 mice.* Aging, 2019. **11**(2): p. 756-770.
18. Chen Scarabelli, C., et al., *Oral administration of amino acidic supplements improves protein and energy profiles in skeletal muscle of aged rats: elongation of functional performance and acceleration of mitochondrial recovery in adenosine triphosphate after exhaustive exertion.* American Journal of Cardiology, 2008. **101**(11A): p. 42E-48E.
19. Crupi, A.N., et al., *Oxidative muscles have better mitochondrial homeostasis than glycolytic muscles throughout life and maintain mitochondrial function during aging.* Aging, 2018. **10**(11): p. 3327-3352.
20. Darvin, K., et al., *Plasma protein biomarkers of the geriatric syndrome of frailty.* Journals of Gerontology Series A-Biological Sciences & Medical Sciences, 2014. **69**(2): p. 182-6.
21. Davizon-Castillo, P., et al., *TNF-a–driven inflammation and mitochondrial dysfunction define the platelet hyperreactivity of aging.* Blood, 2019. **134**(9): p. 727-740.
22. Del Campo, A., et al., *Muscle function decline and mitochondria changes in middle age precede sarcopenia in mice.* Aging, 2018. **10**(1): p. 34-55.
23. Derbre, F., et al., *Age associated low mitochondrial biogenesis may be explained by lack of response of PGC-1alpha to exercise training.* Age, 2012. **34**(3): p. 669-79.
24. DeRuisseau, K.C., et al., *Aging-related changes in the iron status of skeletal muscle.* Experimental Gerontology, 2013. **48**(11): p. 1294-302.
25. Dillon, L.M., et al., *Increased mitochondrial biogenesis in muscle improves aging phenotypes in the mtDNA mutator mouse.* Human Molecular Genetics, 2012. **21**(10): p. 2288-97.
26. Dirks, A. and C. Leeuwenburgh, *Apoptosis in skeletal muscle with aging.* American Journal of Physiology - Regulatory Integrative & Comparative Physiology, 2002. **282**(2): p. R519-27.
27. Dirks, A.J. and C. Leeuwenburgh, *Aging and lifelong calorie restriction result in adaptations of skeletal muscle apoptosis repressor, apoptosis-inducing factor, X-linked inhibitor of apoptosis, caspase-3, and caspase-12.* Free Radic Biol Med, 2004. **36**(1): p. 27-39.
28. Distefano, G., et al., *Physical activity unveils the relationship between mitochondrial energetics, muscle quality and physical function in older adults.* bioRxiv, 2017: p. 164160.
29. Donoghue, P., P. Doran, and K. Ohlendieck, *Biochemical and proteomic profiling of key metabolic enzymes in aging skeletal muscle*, in *Handbook of Nutritional Biochemistry: Genomics, Metabolomics and Food Supply*. 2010. p. 425-443.
30. Drummond, M.J., et al., *Downregulation of E3 ubiquitin ligases and mitophagy-related genes in skeletal muscle of physically inactive, frail older women: A cross-sectional comparison.* Journals of Gerontology - Series A Biological Sciences and Medical Sciences, 2014. **69**(8): p. 1040-1048.
31. Faitg, J., et al., *Effects of Aging and Caloric Restriction on Fiber Type Composition, Mitochondrial Morphology and Dynamics in Rat Oxidative and Glycolytic Muscles.* Frontiers in Physiology, 2019. **10**: p. 420.
32. Figueiredo, P.A., et al., *Impact of lifelong sedentary behavior on mitochondrial function of mice skeletal muscle.* Journals of Gerontology - Series A Biological Sciences and Medical Sciences, 2009. **64**(9): p. 927-939.
33. Fontana, L., et al., *Identification of a metabolic signature for multidimensional impairment and mortality risk in hospitalized older patients.* Aging Cell, 2013. **12**(3): p. 459-66.
34. Fried, L.P., et al., *Nonlinear multisystem physiological dysregulation associated with frailty in older women: Implications for etiology and treatment.* Journals of Gerontology - Series A Biological Sciences and Medical Sciences, 2009. **64**(10): p. 1049-1057.
35. Garcia-Valles, R., et al., *Life-long spontaneous exercise does not prolong lifespan but improves health span in mice.* Longev Healthspan, 2013. **2**(1): p. 14.
36. Gaugler, M., et al., *PKB signaling and atrogene expression in skeletal muscle of aged mice.* Journal of Applied Physiology, 2011. **111**(1): p. 192-199.
37. Gill, J.F., et al., *PGC-1α regulates mitochondrial calcium homeostasis, SR stress and cell death to mitigate skeletal muscle aging.* bioRxiv, 2018: p. 451229.
38. Gram, M., et al., *Skeletal muscle mitochondrial H2 O2 emission increases with immobilization and decreases after aerobic training in young and older men.* Journal of Physiology, 2015. **593**(17): p. 4011-27.
39. Halon-Golabek, M., et al., *hmSOD1 gene mutation-induced disturbance in iron metabolism is mediated by impairment of Akt signalling pathway.* Journal of Cachexia, Sarcopenia and Muscle, 2018. **9**(3): p. 557-569.
40. Hiona, A., et al., *Mitochondrial DNA mutations induce mitochondrial dysfunction, apoptosis and sarcopenia in skeletal muscle of mitochondrial DNA mutator mice.* PLoS ONE [Electronic Resource], 2010. **5**(7): p. e11468.
41. Hofer, T., et al., *Increased iron content and RNA oxidative damage in skeletal muscle with aging and disuse atrophy.* Experimental Gerontology, 2008. **43**(6): p. 563-570.
42. Hong, X., et al., *Relationship between nutritional status and frailty in hospitalized older patients.* Clinical Interventions In Aging, 2019. **14**: p. 105-111.
43. Horii, N., et al., *Resistance training prevents muscle fibrosis and atrophy via down-regulation of C1q-induced Wnt signaling in senescent mice.* FASEB Journal, 2018. **32**(7): p. 3547-3559.
44. Huang, D.D., et al., *Nrf2 deficiency exacerbates frailty and sarcopenia by impairing skeletal muscle mitochondrial biogenesis and dynamics in an age-dependent manner.* Experimental Gerontology, 2019. **119**: p. 61-73.
45. Ibebunjo, C., et al., *Genomic and proteomic profiling reveals reduced mitochondrial function and disruption of the neuromuscular junction driving rat sarcopenia.* Molecular & Cellular Biology, 2013. **33**(2): p. 194-212.
46. Inoue, A., et al., *Exercise restores muscle stem cell mobilization, regenerative capacity and muscle metabolic alterations via adiponectin/AdipoR1 activation in SAMP10 mice.* Journal of Cachexia, Sarcopenia and Muscle, 2017. **8**(3): p. 370-385.
47. Jang, Y.C., et al., *Dietary restriction attenuates age-associated muscle atrophy by lowering oxidative stress in mice even in complete absence of CuZnSOD.* Aging Cell, 2012. **11**(5): p. 770-82.
48. Jang, Y.C., et al., *Increased superoxide in vivo accelerates age-associated muscle atrophy through mitochondrial dysfunction and neuromuscular junction degeneration.* FASEB Journal, 2010. **24**(5): p. 1376-90.
49. Johnson, M.L., et al., *Differential Effect of Endurance Training on Mitochondrial Protein Damage, Degradation, and Acetylation in the Context of Aging.* Journals of Gerontology Series A-Biological Sciences & Medical Sciences, 2015. **70**(11): p. 1386-93.
50. Joseph, A.M., et al., *The impact of aging on mitochondrial function and biogenesis pathways in skeletal muscle of sedentary high- and low-functioning elderly individuals.* Aging Cell, 2012. **11**(5): p. 801-9.
51. Joseph, A.M., et al., *Dysregulation of mitochondrial quality control processes contribute to sarcopenia in a mouse model of premature aging.* PLoS ONE [Electronic Resource], 2013. **8**(7): p. e69327.
52. Kadoguchi, T., et al., *Promotion of oxidative stress is associated with mitochondrial dysfunction and muscle atrophy in aging mice.* Geriatr Gerontol Int, 2019.
53. Kang, C. and W. Lim, *Data on mitochondrial function in skeletal muscle of old mice in response to different exercise intensity.* Data in Brief, 2016. **7**: p. 1519-23.
54. Kishida, Y., et al., *Go-sha-jinki-Gan (GJG), a traditional Japanese herbal medicine, protects against sarcopenia in senescence-accelerated mice.* Phytomedicine, 2015. **22**(1): p. 16-22.
55. Lalia, A.Z., et al., *Influence of omega-3 fatty acids on skeletal muscle protein metabolism and mitochondrial bioenergetics in older adults.* Aging, 2017. **9**(4): p. 1096-1129.
56. Leduc-Gaudet, J.P., et al., *Mitochondrial morphology is altered in atrophied skeletal muscle of aged mice.* Oncotarget, 2015. **6**(20): p. 17923-17937.
57. Leeuwenburgh, C., et al., *Age-related differences in apoptosis with disuse atrophy in soleus muscle.* American Journal of Physiology - Regulatory Integrative and Comparative Physiology, 2005. **288**(5 57-5): p. R1288-R1296.
58. Leng, S., et al., *Serum interleukin-6 and hemoglobin as physiological correlates in the geriatric syndrome of frailty: A pilot study.* Journal of the American Geriatrics Society, 2002. **50**(7): p. 1268-1271.
59. Li, F.H., et al., *Proteomics-based identification of different training adaptations of aged skeletal muscle following long-term high-intensity interval and moderate-intensity continuous training in aged rats.* Aging, 2019. **11**(12): p. 4159-4182.
60. Lirola, E.M.L., M.C.I. Ibabe, and J.M.P. Herreros, *The calf circumference as a quick-reliable marker of malnutrition in hospitalized elderly. Its relation with age and gender.* Nutricion Hospitalaria, 2016. **33**(3): p. 565-571.
61. Ljubicic, V. and D.A. Hood, *Diminished contraction-induced intracellular signaling towards mitochondrial biogenesis in aged skeletal muscle.* Aging Cell, 2009. **8**(4): p. 394-404.
62. Lumbers, M., et al., *Nutritional status in elderly female hip fracture patients: comparison with an age-matched home living group attending day centres.* British Journal of Nutrition, 2001. **85**(6): p. 733-40.
63. Martin, C., et al., *Abnormalities of mitochondrial functioning can partly explain the metabolic disorders encountered in sarcopenic gastrocnemius.* Aging Cell, 2007. **6**(2): p. 165-77.
64. Marzetti, E., et al., *Association between myocyte quality control signaling and sarcopenia in old hip-fractured patients: Results from the Sarcopenia in HIp FracTure (SHIFT) exploratory study.* Experimental Gerontology, 2016. **80**: p. 1-5.
65. Marzetti, E., et al., *Skeletal muscle apoptotic signaling predicts thigh muscle volume and gait speed in community-dwelling older persons: an exploratory study.* PLoS One, 2012. **7**(2): p. e32829.
66. Marzetti, E., et al., *Age-related activation of mitochondrial caspase-independent apoptotic signaling in rat gastrocnemius muscle.* Mechanisms of Ageing & Development, 2008. **129**(9): p. 542-9.
67. Mathieu-Costello, O., et al., *Greater capillary-fiber interface per fiber mitochondrial volume in skeletal muscles of old rats.* Journal of Applied Physiology, 2005. **99**(1): p. 281-289.
68. McMullen, C.A., et al., *Age-related changes of cell death pathways in rat extraocular muscle.* Experimental Gerontology, 2009. **44**(6-7): p. 420-5.
69. Mohamed, J.S., et al., *Dysregulation of SIRT-1 in aging mice increases skeletal muscle fatigue by a PARP-1-dependent mechanism.* Aging, 2014. **6**(10): p. 820-834.
70. Muhammad, M.H. and M.M. Allam, *Resveratrol and/or exercise training counteract aging-associated decline of physical endurance in aged mice; targeting mitochondrial biogenesis and function.* Journal of Physiological Sciences: JPS, 2018. **68**(5): p. 681-688.
71. Myers, M.J., et al., *The role of SIRT1 in skeletal muscle function and repair of older mice.* Journal of Cachexia, Sarcopenia and Muscle, 2019. **10**(4): p. 929-949.
72. O'Connell, K. and K. Ohlendieck, *Proteomic DIGE analysis of the mitochondria-enriched fraction from aged rat skeletal muscle.* Proteomics, 2009. **9**(24): p. 5509-24.
73. Ogata, T., et al., *Differential cell death regulation between adult-unloaded and aged rat soleus muscle.* Mechanisms of Ageing & Development, 2009. **130**(5): p. 328-36.
74. Pasini, E., et al., *Effects of treadmill exercise and training frequency on anabolic signaling pathways in the skeletal muscle of aged rats.* Experimental Gerontology, 2012. **47**(1): p. 23-8.
75. Pestronk, A., R. Keeling, and R. Choksi, *Sarcopenia, age, atrophy, and myopathy: Mitochondrial oxidative enzyme activities.* Muscle & Nerve, 2017. **56**(1): p. 122-128.
76. Picard, M., et al., *Alterations in intrinsic mitochondrial function with aging are fiber type-specific and do not explain differential atrophy between muscles.* Aging Cell, 2011. **10**(6): p. 1047-1055.
77. Pietrangelo, L., et al., *Age-dependent uncoupling of mitochondria from Ca2+ release units in skeletal muscle.* Oncotarget, 2015. **6**(34): p. 35358-71.
78. Rice, K.M. and E.R. Blough, *Sarcopenia-related apoptosis is regulated differently in fast- and slow-twitch muscles of the aging F344/N x BN rat model.* Mechanisms of Ageing & Development, 2006. **127**(8): p. 670-9.
79. Rodriguez-Bies, E., et al., *Resveratrol primes the effects of physical activity in old mice.* British Journal of Nutrition, 2016. **116**(6): p. 979-88.
80. Ryan, M.J., et al., *Inhibition of xanthine oxidase reduces oxidative stress and improves skeletal muscle function in response to electrically stimulated isometric contractions in aged mice.* Free Radical Biology and Medicine, 2011. **51**(1): p. 38-52.
81. Safdar, A., et al., *Endurance exercise rescues progeroid aging and induces systemic mitochondrial rejuvenation in mtDNA mutator mice.* Proceedings of the National Academy of Sciences of the United States of America, 2011. **108**(10): p. 4135-4140.
82. Safdar, A., et al., *Aberrant mitochondrial homeostasis in the skeletal muscle of sedentary older adults.* PLoS ONE [Electronic Resource], 2010. **5**(5): p. e10778.
83. Shah, V.O., et al., *Mitochondrial DNA deletion and sarcopenia.* Genet Med, 2009. **11**(3): p. 147-52.
84. Siegel, M.P., et al., *Mitochondrial-targeted peptide rapidly improves mitochondrial energetics and skeletal muscle performance in aged mice.* Aging Cell, 2013. **12**(5): p. 763-71.
85. Silva, J.C., et al., *Understanding red blood cell parameters in the context of the frailty phenotype: Interpretations of the FIBRA (Frailty in Brazilian Seniors) study.* Archives of Gerontology and Geriatrics, 2014. **59**(3): p. 636-641.
86. Siu, P.M., E.E. Pistilli, and S.E. Alway, *Apoptotic responses to hindlimb suspension in gastrocnemius muscles from young adult and aged rats.* American Journal of Physiology - Regulatory Integrative and Comparative Physiology, 2005. **289**(4 58-4): p. R1015-R1026.
87. Sonjak, V., et al., *Reduced Mitochondrial Content, Elevated Reactive Oxygen Species, and Modulation by Denervation in Skeletal Muscle of Prefrail or Frail Elderly Women.* J Gerontol A Biol Sci Med Sci, 2019. **74**(12): p. 1887-1895.
88. Spendiff, S., et al., *Denervation drives mitochondrial dysfunction in skeletal muscle of octogenarians.* Journal of Physiology, 2016. **594**(24): p. 7361-7379.
89. St-Jean-Pelletier, F., et al., *The impact of ageing, physical activity, and pre-frailty on skeletal muscle phenotype, mitochondrial content, and intramyocellular lipids in men.* J Cachexia Sarcopenia Muscle, 2017. **8**(2): p. 213-228.
90. Szczesny, B., A.W. Tann, and S. Mitra, *Age- and tissue-specific changes in mitochondrial and nuclear DNA base excision repair activity in mice: Susceptibility of skeletal muscles to oxidative injury.* Mechanisms of Ageing and Development, 2010. **131**(5): p. 330-337.
91. Tamura, Y., et al., *Effects of Heat Stress Treatment on Age-dependent Unfolded Protein Response in Different Types of Skeletal Muscle.* J Gerontol A Biol Sci Med Sci, 2017. **72**(3): p. 299-308.
92. Tezze, C., et al., *Age-Associated Loss of OPA1 in Muscle Impacts Muscle Mass, Metabolic Homeostasis, Systemic Inflammation, and Epithelial Senescence.* Cell Metabolism, 2017. **25**(6): p. 1374-1389.e6.
93. Théron, L., et al., *Label-free quantitative protein profiling of vastus lateralis muscle during human aging.* Molecular and Cellular Proteomics, 2014. **13**(1): p. 283-294.
94. Vettoretti, S., et al., *Sarcopenia is associated with malnutrition but not with systemic inflammation in older persons with advanced CKD.* Nutrients, 2019. **11**(6).
95. Walsh, M.E., et al., *The histone deacetylase inhibitor butyrate improves metabolism and reduces muscle atrophy during aging.* Aging Cell, 2015. **14**(6): p. 957-70.
96. Wang, X., et al., *Transient systemic mtDNA damage leads to muscle wasting by reducing the satellite cell pool.* Hum Mol Genet, 2013. **22**(19): p. 3976-86.
97. Wang, X.X., et al., *ERR agonism reverses mitochondrial dysfunction and inflammation in the aging kidney.* bioRxiv, 2019: p. 755801.
98. Waters, D.L., et al., *Mitochondrial function in physically active elders with sarcopenia.* Mech Ageing Dev, 2009. **130**(5): p. 315-9.
99. Wenz, T., et al., *Increased muscle PGC-1α expression protects from sarcopenia and metabolic disease during aging.* Proceedings of the National Academy of Sciences, 2009. **106**(48): p. 20405-20410.
100. Westbrook, R.M., et al., *Aged interleukin-10tm1Cgn chronically inflamed mice have substantially reduced fat mass, metabolic rate, and adipokines.* PLoS ONE, 2017. **12**(12).
101. White, Z., et al., *Voluntary resistance wheel exercise from mid-life prevents sarcopenia and increases markers of mitochondrial function and autophagy in muscles of old male and female C57BL/6J mice.* Skelet Muscle, 2016. **6**(1): p. 45.
102. Wu, C., et al., *A persistent level of CISD2 extends healthy lifespan and delays aging in mice.* Human Molecular Genetics, 2012. **21**(18): p. 3956-3968.
103. Xu, J., et al., *Long-term perturbation of muscle iron homeostasis following hindlimb suspension in old rats is associated with high levels of oxidative stress and impaired recovery from atrophy.* Experimental Gerontology, 2012. **47**(1): p. 100-108.
104. Xu, J., et al., *Iron accumulation with age, oxidative stress and functional decline.* PLoS One, 2008. **3**(8): p. e2865.
105. Yeo, D., et al., *Intensified mitophagy in skeletal muscle with aging is downregulated by PGC-1alpha overexpression in vivo.* Free Radic Biol Med, 2019. **130**: p. 361-368.
106. Zangarelli, A., et al., *Synergistic effects of caloric restriction with maintained protein intake on skeletal muscle performance in 21-month-old rats: a mitochondria-mediated pathway.* Faseb j, 2006. **20**(14): p. 2439-50.
107. Zhang, X., et al., *Metabolite profile and mitochondrial energetics characterize poor early recovery of muscle mass following hind limb unloading in old mice.* bioRxiv, 2017: p. 183244.
