# Supplementary material for: A systematic review and meta‐analysis of studies that have evaluated the role of mitochondrial function and iron metabolism in frailty
Source: Clin Transl Sci. 2021 Jul 9;14(6):2370–8. doi: 10.1111/cts.13101 (PMC8604243; doi:10.1111/cts.13101)
Supplement: Supplementary file 5 — File S5 [file CTS-14-2370-s003.docx]

**Table S2: Mitochondrial morphology**

| ***Outcome*** | ***Primary analysis*** | | | | | | ***Heterogeneity analysis*** | | | | ***Subgroup analysis*** | | | | | |
| --- | --- | --- | --- | --- | --- | --- | --- | --- | --- | --- | --- | --- | --- | --- | --- | --- |
|  | **No. of studies** | **Estimate** | **p value** | **Lower CI** | **Upper CI** | **I^2** | **Type of heterogeneity** | **QM p value** | **No. of studies per source of heterogeneity** | | **Estimate** | **p value** | **Lower CI** | **Upper CI** | **I^2** | **Qp value** |
| **Mitochondria number** | 4 | -2.63 | 0.105 | -5.80 | 0.55 | 91% | Species | NA | Mouse | 4 | -2.63 | 0.110 | -5.80 | 0.55 | 91% | 0.000 |
|  |  |  |  |  |  |  | Frailty assessment | 0.394 | Assumed based on age | 3 | -2.89 | 0.220 | -7.49 | 1.71 | 93% | 0.000 |
|  |  |  |  |  |  |  |  |  | Genetically modified models | 1 | -2.32 | 0.011 | -4.11 | -0.53 | 0% | 1.000 |
|  | | |  |  |  |  | | | | | | | | | | |
| **Mitochondrial size** | 3 | -0.71 | 0.613 | -3.48 | 2.05 | 87% | Species | 0.670 | Rat | 1 | -2.47 | 0.008 | -4.31 | -0.63 | 0% | 1.000 |
|  |  |  |  |  |  |  |  |  | Mouse | 2 | 0.17 | 0.929 | -3.65 | 3.99 | 89% | 0.002 |
|  |  |  |  |  |  |  | Frailty assessment | NA | Assumed based on age | 3 | -0.71 | 0.613 | -3.48 | 2.05 | 86% | 0.002 |
|  | | |  |  |  |  | | | | | | | | | | |
| **Mitochondrial volume density** | 5 | -0.38 | 0.227 | -0.99 | 0.23 | 0% | Species | 0.576 | Rat | 2 | -0.43 | 0.306 | -1.24 | 0.39 | 0% | 0.438 |
|  |  |  |  |  |  |  |  |  | Mouse | 3 | -2.30 | 0.376 | -7.39 | 2.79 | 95% | 0.002 |
|  |  |  |  |  |  |  | Frailty assessment | NA | Assumed based on age | 5 | -0.38 | 0.227 | -0.99 | 0.24 | 0% | 0.013 |
|  | | | | | | | | | | | | | | | | |
| **mtDNA copy number** | 4 | -2.39 | 0.072 | -5.0 | 0.21 | 97% | Species | 0.042 | Naked mole rat | 1 | 0.35 | 0.11 | -6.00 | -0.74 | 0% | 1.000 |
|  |  |  |  |  |  |  |  |  | Mouse | 3 | -3.37 | 0.012 | -6.00 | -0.74 | 84% | 0.000 |
|  |  |  |  |  |  |  | Frailty assessment | 0.042 | Assumed based on age | 1 | 0.35 | 0.11 | -0.08 | 0.77 | 0% | 1.000 |
|  |  |  |  |  |  |  |  |  | Genetically modified models | 3 | -3.37 | 0.012 | -6.00 | -0.74 | 84% | 0.000 |
|  | | | | | | | | | | | | | | | | |
| **mtDNA/nuclear DNA ratio** | 3 | -1.61 | 0.021 | -2.97 | -0.25 | 57% | Species | 0.004 | Human | 1 | -0.77 | 0.082 | -1.64 | 0.10 | 0% | 1.000 |
|  |  |  |  |  |  |  |  |  | Mouse | 2 | -2.33 | 0.002 | -3.83 | -0.84 | 14% | 0.280 |
|  |  |  |  |  |  |  | Frailty assessment | NA | Assumed based on age | 1 | -3.18 | 0.003 | -5.26 | -1.09 | 0% | 1.000 |
|  |  |  |  |  |  |  |  |  | Fried frailty index | 1 | -0.77 | 0.082 | -1.64 | 0.10 | 0% | 1.000 |
|  |  |  |  |  |  |  |  |  | Genetically modified models | 1 | -1.64 | 0.082 | -3.49 | 0.21 | 0% | 1.000 |
|  | | | | | | | | | | | | | | | | |
| **VDAC protein expression** | 5 | 0.01 | 0.988 | -0.94 | 0.96 | 73% | Species | 0.438 | Human | 2 | -0.44 | 0.583 | -2.03 | 1.14 | 85% | 0.009 |
|  |  |  |  |  |  |  |  |  | Monkey | 1 | -0.79 | 0.282 | -2.23 | 0.65 | 0% | 1.000 |
|  |  |  |  |  |  |  |  |  | Mouse | 2 | 0.99 | 0.038 | 0.06 | 1.93 | 0% | 0.432 |
|  |  |  |  |  |  |  | Frailty assessment | 0.265 | Assumed based on age | 2 | -0.10 | 0.878 | -1.44 | 1.23 | 43% | 0.187 |
|  |  |  |  |  |  |  |  |  | Immobilisation/ sedentary lifestyle | 1 | 0.39 | 0.417 | -0.55 | 1.33 | 0% | 1.000 |
|  |  |  |  |  |  |  |  |  | Fried frailty index | 1 | -1.23 | 0.002 | -1.98 | -0.47 | 0% | 1.000 |
|  |  |  |  |  |  |  |  |  | Genetically modified models | 1 | 1.33 | 0.038 | 0.08 | 2.58 | 0% | 1.000 |

| ***Outcome*** | ***Primary analysis*** | | | | | | ***Heterogeneity analysis*** | | | | | ***Subgroup analysis*** | | | | | |
| --- | --- | --- | --- | --- | --- | --- | --- | --- | --- | --- | --- | --- | --- | --- | --- | --- | --- |
|  | **No. of studies** | **Estimate** | **p value** | **Lower CI** | **Upper CI** | **I^2** | **Type of heterogeneity** | **QM p value** | **No. of studies per source of heterogeneity** | | **Estimate** | | **p value** | **Lower CI** | **Upper CI** | **I^2** | **Qp value** |
| **DRP1 gene expression** | 6 | -4.74 | 0.208 | -12.10 | 2.63 | 99% | Species | 0.000 | Human | 2 | -1.37 | | 0.001 | -2.16 | -0.60 | 0% | 0.733 |
|  |  |  |  |  |  |  |  |  | Rat | 1 | -31.37 | | 0.000 | -43.98 | -18.77 | 0% | 1.000 |
|  |  |  |  |  |  |  |  |  | Mouse | 3 | -0.97 | | 0.173 | -2.37 | 0.43 | 60% | 0.075 |
|  |  |  |  |  |  |  | Frailty assessment | 0.716 | Assumed based on age | 2 | -15.66 | | 0.297 | -45.11 | 13.79 | 95% | 0.000 |
|  |  |  |  |  |  |  |  |  | Immobilisation/ sedentary lifestyle | 1 | -1.51 | | 0.006 | -2.57 | -0.44 | 0% | 1.000 |
|  |  |  |  |  |  |  |  |  | Other geriatric assessment | 1 | -1.23 | | 0.034 | -2.38 | -0.09 | 0% | 1.000 |
|  |  |  |  |  |  |  |  |  | Genetically modified models | 2 | -0.91 | | 0.422 | -3.15 | 1.32 | 78% | 0.031 |
|  | | | | | | | | | | | | | | | | | |
| **DRP1 protein expression** | 7 | -0.06 | 0.913 | -1.15 | 1.03 | 81% | Species | 0.768 | Human | 1 | 0.24 | | 0.561 | -0.58 | 1.07 | 0% | 1.000 |
|  |  |  |  |  |  |  |  |  | Rat | 1 | 1.36 | | 0.038 | 0.07 | 2.65 | 0% | 1.000 |
|  |  |  |  |  |  |  |  |  | Mouse | 5 | -0.41 | | 0.579 | -1.84 | 1.03 | 80% | 0.000 |
|  |  |  |  |  |  |  | Frailty assessment | 0.791 | Assumed based on age | 4 | 1.01 | | 0.410 | -1.39 | 3.41 | 89% | 0.000 |
|  |  |  |  |  |  |  |  |  | Other geriatric assessment | 1 | 0.24 | | 0.561 | -0.58 | 1.07 | 0% | 1.000 |
|  |  |  |  |  |  |  |  |  | Genetically modified models | 2 | -0.79 | | 0.053 | -1.59 | 0.01 | 8% | 0.295 |
|  | | | | | | | | | | | | | | | | | |
| **FIS1 proteins expression** | 5 | 2.00 | 0.081 | -0.25 | 4.24 | 94% | Species | 0.000 | Human | 2 | 0.45 | | 0.122 | -0.12 | 1.04 | 0% | 0.479 |
|  |  |  |  |  |  |  |  |  | Rat | 1 | -0.25 | | 0.677 | -1.43 | 0.93 | 0% | 1.000 |
|  |  |  |  |  |  |  |  |  | Mouse | 2 | 4.99 | | 0.000 | 3.63 | 6.36 | 0% | 0.716 |
|  |  |  |  |  |  |  | Frailty assessment | 0.560 | Sarcopenia/ muscle atrophy | 1 | 0.67 | | 0.111 | -0.15 | 1.49 | 0% | 1.000 |
|  |  |  |  |  |  |  |  |  | Assumed based on age | 2 | 2.16 | | 0.384 | -2.70 | 7.02 | 94% | 0.000 |
|  |  |  |  |  |  |  |  |  | Other geriatric assessment | 1 | 0.25 | | 0.554 | -0.57 | 1.07 | 0% | 1.000 |
|  |  |  |  |  |  |  |  |  | Genetically modified models | 1 | 5.22 | | 0.000 | 3.38 | 7.06 | 0% | 1.000 |
|  | | | | | | | | | | | | | | | | | |
| **Mfn1 gene expression** | 5 | -4.71 | 0.147 | -11.06 | 1.65 | 98% | Species | 0.000 | Human | 1 | -1.65 | | 0.003 | -2.74 | -0.56 | 0% | 1.000 |
|  |  |  |  |  |  |  |  |  | Rat | 1 | -21.59 | | 0.000 | -30.31 | -12.88 | 0% | 1.000 |
|  |  |  |  |  |  |  |  |  | Mouse | 3 | -1.56 | | 0.001 | -2.45 | -0.67 | 0% | 0.441 |
|  |  |  |  |  |  |  | Frailty assessment | 0.524 | Assumed based on age | 2 | -10.83 | | 0.292 | -31.00 | 9.34 | 95% | 0.000 |
|  |  |  |  |  |  |  |  |  | Immobilisation/ sedentary lifestyle | 1 | -1.65 | | 0.003 | -2.74 | -0.56 | 0% | 1.000 |
|  |  |  |  |  |  |  |  |  | Genetically modified models | 2 | -1.79 | | 0.001 | -2.86 | -0.71 | 4% | 0.308 |

**Table S3: Mitochondrial dynamics**

**Continued on the next page**

| ***Outcome*** | ***Primary analysis*** | | | | | | ***Heterogeneity analysis*** | | | | ***Subgroup analysis*** | | | | | |
| --- | --- | --- | --- | --- | --- | --- | --- | --- | --- | --- | --- | --- | --- | --- | --- | --- |
|  | **No. of studies** | **Estimate** | **p value** | **Lower CI** | **Upper CI** | **I^2** | **Type of heterogeneity** | **QM p value** | **No. of studies per source of heterogeneity** | | **Estimate** | **p value** | **Lower CI** | **Upper CI** | **I^2** | **Qp value** |
| **Mfn1 protein expression** | 3 | 0.33 | 0.227 | -0.20 | 0.85 | 0% | Species | 0.468 | Human | 1 | 0.40 | 0.332 | -0.41 | 1.21 | 0% | 1.000 |
|  |  |  |  |  |  |  |  |  | Mouse | 2 | 0.27 | 0.448 | -0.43 | 0.97 | 0% | 0.790 |
|  |  |  |  |  |  |  | Frailty assessment | NA | Sarcopenia/ muscle atrophy | 1 | 0.40 | 0.332 | -0.42 | 1.21 | 0% | 1.000 |
|  |  |  |  |  |  |  |  |  | Assumed based on age | 1 | 0.39 | 0.501 | -0.75 | 1.54 | 0% | 1.000 |
|  |  |  |  |  |  |  |  |  | Genetically modified models | 1 | 0.20 | 0.660 | -0.68 | 1.08 | 0% | 1.000 |
|  |  |  |  |  |  |  |  |  |  |  |  |  |  |  |  |  |
| **Mfn2 gene expression** | 8 | -2.08 | 0.206 | -5.31 | 1.14 | 97% | Species | 0.000 | Human | 2 | -1.31 | 0.047 | -2.60 | -0.02 | 63% | 0.100 |
|  |  |  |  |  |  |  |  |  | Rat | 1 | -21.89 | 0.000 | -30.71 | -13.06 | 0% | 1.000 |
|  |  |  |  |  |  |  |  |  | Mouse | 6 | -0.27 | 0.745 | -1.92 | 1.38 | 83% | 0.001 |
|  |  |  |  |  |  |  | Frailty assessment | 0.883 | Assumed based on age | 4 | -4.64 | 0.360 | -14.59 | 5.31 | 99% | 0.000 |
|  |  |  |  |  |  |  |  |  | Immobilisation/ sedentary lifestyle | 1 | -1.99 | 0.001 | -3.14 | -0.84 | 0% | 1.000 |
|  |  |  |  |  |  |  |  |  | Other geriatric assessment | 1 | -0.67 | 0.224 | -1.74 | 0.41 | 0% | 1.000 |
|  |  |  |  |  |  |  |  |  | Genetically modified models | 2 | -1.10 | 0.507 | -4.33 | 2.14 | 88% | 0.004 |
|  | | | | | | | | | | | | | | | | |
| **Mfn2 protein expression** | 6 | 0.01 | 0.978 | -0.93 | 0.95 | 80% | Species | 0.916 | Human | 2 | -0.35 | 0.516 | -1.40 | 0.70 | 69% | 0.073 |
|  |  |  |  |  |  |  |  |  | Rat | 1 | 0.89 | 0.155 | -0.34 | 2.12 | 0% | 1.000 |
|  |  |  |  |  |  |  |  |  | Mouse | 3 | 0.02 | 0.986 | -1.86 | 1.90 | 88% | 0.000 |
|  |  |  |  |  |  |  | Frailty assessment | 0.000 | Sarcopenia/ muscle atrophy | 1 | -0.89 | 0.038 | -1.73 | -0.05 | 0% | 1.000 |
|  |  |  |  |  |  |  |  |  | Assumed based on age | 3 | 0.95 | 0.006 | 0.27 | 1.63 | 0% | 0.833 |
|  |  |  |  |  |  |  |  |  | Other geriatric assessment | 1 | 0.18 | 0.659 | -0.64 | 1.00 | 0% | 1.000 |
|  |  |  |  |  |  |  |  |  | Genetically modified models | 1 | -1.85 | 0.001 | -2.90 | -0.81 | 0% | 1.000 |
|  | | | | | | | | | | | | | | | | |
| **Opa1 gene expression** | 5 | -4.71 | 0.128 | -10.79 | 1.36 | 98% | Species | 0.000 | Human | 1 | -1.78 | 0.002 | -2.89 | -0.67 | 0% | 1.000 |
|  |  |  |  |  |  |  |  |  | Rat | 1 | -20.43 | 0.000 | -28.68 | -12.18 | 0% | 1.000 |
|  |  |  |  |  |  |  |  |  | Mouse | 3 | -1.67 | 0.130 | -3.84 | 0.49 | 78% | 0.009 |
|  |  |  |  |  |  |  | Frailty assessment | 0.289 | Assumed based on age | 2 | -11.54 | 0.169 | -28.00 | 4.91 | 93% | 0.000 |
|  |  |  |  |  |  |  |  |  | Immobilisation/ sedentary lifestyle | 1 | -1.78 | 0.002 | -2.89 | -0.67 | 0% | 1.000 |
|  |  |  |  |  |  |  |  |  | Genetically modified models | 2 | -0.94 | 0.421 | -3.23 | 1.35 | 79% | 0.028 |
|  | | | | | | | | | | | | | | | | |
| **Opa1 protein expression** | 7 | 0.14 | 0.715 | -0.63 | 0.92 | 73% | Species | 0.179 | Human | 2 | -0.68 | 0.023 | -2.89 | -0.09 | 0% | 0.610 |
|  |  |  |  |  |  |  |  |  | Mouse | 5 | 0.55 | 0.247 | -28.68 | 1.48 | 67% | 0.026 |
|  |  |  |  |  |  |  | Frailty assessment | 0.359 | Sarcopenia/ muscle atrophy | 1 | -0.54 | 0.197 | -3.84 | 0.28 | 0% | 1.000 |
|  |  |  |  |  |  |  |  |  | Assumed based on age | 3 | 1.04 | 0.027 | -28.00 | 1.96 | 35% | 0.222 |
|  |  |  |  |  |  |  |  |  | Other geriatric assessment | 1 | -0.84 | 0.053 | -2.89 | 0.01 | 0% | 1.000 |
|  |  |  |  |  |  |  |  |  | Genetically modified models | 2 | -0.20 | 0.828 | -3.23 | 1.62 | 80% | 0.024 |

**Table S3 (continued): Mitochondrial dynamics**

**Table S4: Mitochondrial respiratory chain complexes**

| ***Outcome*** | ***Primary analysis*** | | | | | | ***Heterogeneity analysis*** | | | | ***Subgroup analysis*** | | | | | |
| --- | --- | --- | --- | --- | --- | --- | --- | --- | --- | --- | --- | --- | --- | --- | --- | --- |
|  | **No. of studies** | **Estimate** | **p value** | **Lower CI** | **Upper CI** | **I^2** | **Type of heterogeneity** | **QM p value** | **No. of studies per source of heterogeneity** | | **Estimate** | **p value** | **Lower CI** | **Upper CI** | **I^2** | **Qp value** |
| **Activity of respiratory complex I** | 11 | -0.72 | 0.019 | -1.33 | -0.12 | 73% | Species | 0.150 | Human | 3 | -0.46 | 0.233 | -1.21 | 0.29 | 64% | 0.060 |
|  |  |  |  |  |  |  |  |  | Rat | 2 | -0.48 | 0.760 | -3.57 | 2.61 | 90% | 0.001 |
|  |  |  |  |  |  |  |  |  | Mouse | 6 | -1.00 | 0.024 | -1.87 | -0.13 | 68% | 0.006 |
|  |  |  |  |  |  |  | Frailty assessment | 0.240 | Sarcopenia/ muscle atrophy | 2 | -0.33 | 0.595 | -1.56 | 0.90 | 82% | 0.020 |
|  |  |  |  |  |  |  |  |  | Assumed based on age | 5 | -0.95 | 0.150 | -2.24 | 0.34 | 78% | 0.004 |
|  |  |  |  |  |  |  |  |  | Immobilisation/ sedentary lifestyle | 1 | 0.25 | 0.480 | -0.45 | 0.95 | 0% | 1.000 |
|  |  |  |  |  |  |  |  |  | Fried frailty index | 1 | -0.68 | 0.122 | -1.54 | 0.18 | 0% | 1.000 |
|  |  |  |  |  |  |  |  |  | Genetically modified models | 2 | -1.69 | 0.001 | -2.68 | -0.71 | 0% | 0.349 |
|  | | | | | | | | | | | | | | | | |
| **Activity of respiratory complex II** | 5 | -1.57 | 0.052 | -3.16 | 0.02 | 92% | Species | 0.000 | Human | 2 | -1.26 | 0.000 | -1.83 | -0.68 | 0% | 0.56 |
|  |  |  |  |  |  |  |  |  | Rat | 1 | -6.06 | 0.000 | -8.73 | -3.38 | 0% | 1.000 |
|  |  |  |  |  |  |  |  |  | Mouse | 2 | -0.42 | 0.224 | -1.10 | 0.26 | 0% | 0.640 |
|  |  |  |  |  |  |  | Frailty assessment | 0.857 | Sarcopenia/ muscle atrophy | 1 | -1.40 | 0.000 | -2.15 | -0.65 | 0% | 1.000 |
|  |  |  |  |  |  |  |  |  | Assumed based on age | 2 | -3.03 | 0.293 | -8.68 | 2.62 | 94% | 0.000 |
|  |  |  |  |  |  |  |  |  | Fried frailty index | 1 | -1.05 | 0.021 | -1.94 | -0.16 | 0% | 1.000 |
|  |  |  |  |  |  |  |  |  | Genetically modified models | 1 | -0.62 | 0.256 | -1.70 | 0.45 | 0% | 1.000 |
|  | | | | | | | | | | | | | | | | |
| **Activity of respiratory complex III** | 4 | -0.58 | 0.014 | -1.04 | -0.12 | 0% | Species | 0.048 | Human | 1 | -0.62 | 0.064 | -1.27 | 0.04 | 0% | 1.000 |
|  |  |  |  |  |  |  |  |  | Mouse | 3 | -0.54 | 0.104 | -1.19 | 0.11 | 0% | 0.780 |
|  |  |  |  |  |  |  | Frailty assessment | 0.103 | Sarcopenia/ muscle atrophy | 1 | -0.62 | 0.064 | -1.27 | 0.04 | 0% | 1.000 |
|  |  |  |  |  |  |  |  |  | Assumed based on age | 2 | -0.48 | 0.197 | -1.21 | 0.25 | 0% | 0.541 |
|  |  |  |  |  |  |  |  |  | Genetically modified models | 1 | -0.77 | 0.295 | -2.20 | 0.67 | 0% | 1.000 |
|  | | | | | | | | | | | | | | | | |
| **Activity of respiratory complex IV** | 20 | -0.95 | 0.004 | -1.59 | -0.31 | 87% | Species | 0.085 | Human | 4 | -0.45 | 0.333 | -1.37 | 0.46 | 80% | 0.004 |
|  |  |  |  |  |  |  |  |  | Monkey | 1 | -0.57 | 0.496 | -2.20 | 1.07 | 0% | 1.000 |
|  |  |  |  |  |  |  |  |  | Rat | 8 | -1.15 | 0.059 | -2.34 | 0.04 | 91% | 0.000 |
|  |  |  |  |  |  |  |  |  | Mouse | 7 | -1.19 | 0.071 | -2.48 | 0.10 | 85% | 0.000 |
|  |  |  |  |  |  |  | Frailty assessment | 0.068 | Sarcopenia/ muscle atrophy | 2 | 0.20 | 0.585 | -0.52 | 0.91 | 46% | 0.173 |
|  |  |  |  |  |  |  |  |  | Assumed based on age | 11 | -1.21 | 0.006 | -2.08 | -0.34 | 82% | 0.000 |
|  |  |  |  |  |  |  |  |  | Immobilisation/ sedentary lifestyle | 2 | -0.16 | 0.786 | -1.30 | 0.98 | 79% | 0.030 |
|  |  |  |  |  |  |  |  |  | Fried frailty index | 1 | -1.64 | 0.001 | -2.60 | -0.67 | 0% | 1.000 |
|  |  |  |  |  |  |  |  |  | Genetically modified models | 4 | -1.30 | 0.244 | -3.49 | 0.89 | 91% | 0.000 |

**Continued on the next page**

**Table S4 (continued): Mitochondrial respiratory chain complexes**

| ***Outcome*** | ***Primary analysis*** | | | | | | ***Heterogeneity analysis*** | | | | ***Subgroup analysis*** | | | | | |
| --- | --- | --- | --- | --- | --- | --- | --- | --- | --- | --- | --- | --- | --- | --- | --- | --- |
|  | **No. of studies** | **Estimate** | **p value** | **Lower CI** | **Upper CI** | **I^2** | **Type of heterogeneity** | **QM p value** | **No. of studies per source of heterogeneity** | | **Estimate** | **p value** | **Lower CI** | **Upper CI** | **I^2** | **Qp value** |
| **Activity of respiratory complex V** | 3 | -1.12 | 0.123 | -2.54 | 0.30 | 68% | Species | NA | Mouse | 3 | -1.12 | 0.123 | -2.54 | 0.30 | 68% | 0.064 |
|  |  |  |  |  |  |  |  |  | Assumed based on age | 2 | -1.73 | 0.116 | -3.88 | 0.43 | 70% | 0.069 |
|  |  |  |  |  |  |  | Frailty assessment | 0.285 | Genetically modified models | 1 | -0.26 | 0.623 | -1.32 | 0.79 | 0% | 1.000 |
|  | | | | | | | | | | | | | | | | |
| **Complex I gene expression** | 3 | -7.24 | 0.003 | -12.06 | -2.43 | 84% | Species | 0.000 | Rat | 1 | -13.21 | 0.000 | -18.61 | -7.80 | 0% | 1.000 |
|  |  |  |  |  |  |  |  |  | Mouse | 2 | -4.93 | 0.000 | -6.79 | -3.07 | 0% | 0.657 |
|  |  |  |  |  |  |  | Frailty assessment | 0.044 | Assumed based on age | 2 | -9.00 | 0.019 | -16.55 | -1.45 | 83% | 0.014 |
|  |  |  |  |  |  |  |  |  | Genetically modified models | 1 | -4.60 | 0.000 | -6.97 | -2.24 | 0% | 1.000 |
|  | | | | | | | | | | | | | | | | |
| **Complex II protein expression** | 14 | **-**1.02 | 0.076 | -2.15 | 0.11 | 91% | Species | 0.393 | Human | 3 | -1.40 | 0.001 | -2.22 | -0.58 | 51% | 0.128 |
|  |  |  |  |  |  |  |  |  | Rat | 4 | -1.50 | 0.474 | -5.60 | 2.60 | 96% | 0.000 |
|  |  |  |  |  |  |  |  |  | Mouse | 7 | -0.35 | 0.317 | -1.02 | 0.33 | 45% | 0.010 |
|  |  |  |  |  |  |  | Frailty assessment | 0.094 | Assumed based on age | 6 | 0.17 | 0.799 | -1.16 | 1.51 | 85% | 0.000 |
|  |  |  |  |  |  |  |  |  | Immobilisation/ sedentary lifestyle | 2 | -3.72 | 0.242 | -9.95 | 2.51 | 95% | 0.000 |
|  |  |  |  |  |  |  |  |  | Fried frailty index | 2 | -1.81 | 0.000 | -2.53 | -1.10 | 0% | 0.464 |
|  |  |  |  |  |  |  |  |  | Genetically modified models | 4 | -1.32 | 0.184 | -3.26 | 0.62 | 83% | 0.006 |
|  | | | | | | | | | | | | | | | | |
| **Complex III protein expression** | 8 | -1.44 | 0.000 | -2.25 | -0.64 | 71% | Species | 0.005 | Human | 2 | -1.44 | 0.161 | -3.44 | 0.57 | 88% | 0.003 |
|  |  |  |  |  |  |  |  |  | Mouse | 6 | -1.45 | 0.004 | -2.43 | -0.48 | 68% | 0.011 |
|  |  |  |  |  |  |  | Frailty assessment | 0.031 | Assumed based on age | 3 | -1.47 | 0.137 | -3.41 | 0.47 | 85% | 0.002 |
|  |  |  |  |  |  |  |  |  | Immobilisation/ sedentary lifestyle | 1 | -0.42 | 0.382 | -1.36 | 0.52 | 0% | 1.000 |
|  |  |  |  |  |  |  |  |  | Fried frailty index | 1 | -2.47 | 0.000 | -3.46 | -1.47 | 0% | 1.000 |
|  |  |  |  |  |  |  |  |  | Genetically modified models | 3 | -1.44 | 0.002 | -2.36 | -0.52 | 19% | 0.272 |

**Continued on the next page**

| ***Outcome*** | ***Primary analysis*** | | | | | | ***Heterogeneity analysis*** | | | | ***Subgroup analysis*** | | | | | |
| --- | --- | --- | --- | --- | --- | --- | --- | --- | --- | --- | --- | --- | --- | --- | --- | --- |
|  | **No. of studies** | **Estimate** | **p value** | **Lower CI** | **Upper CI** | **I^2** | **Type of heterogeneity** | **QM p value** | **No. of studies per source of heterogeneity** | | **Estimate** | **p value** | **Lower CI** | **Upper CI** | **I^2** | **Qp value** |
| **Complex IV protein expression** | 15 | -1.10 | 0.008 | -1.91 | -0.28 | 85% | Species | 0.033 | Human | 5 | -0.83 | 0.148 | -1.95 | 0.29 | 86% | 0.000 |
|  |  |  |  |  |  |  |  |  | Monkey | 1 | -0.47 | 0.530 | -1.91 | 0.99 | 0% | 1.000 |
|  |  |  |  |  |  |  |  |  | Rat | 2 | -3.21 | 0.025 | -6.01 | -0.41 | 82% | 0.017 |
|  |  |  |  |  |  |  |  |  | Mouse | 7 | -0.84 | 0.206 | -2.15 | 0.46 | 83% | 0.000 |
|  |  |  |  |  |  |  | Frailty assessment | 0.123 | Assumed based on age | 6 | -0.61 | 0.429 | -2.10 | 0.90 | 86% | 0.000 |
|  |  |  |  |  |  |  |  |  | Immobilisation/ sedentary lifestyle | 3 | -1.96 | 0.186 | -4.85 | 0.95 | 94% | 0.000 |
|  |  |  |  |  |  |  |  |  | Fried frailty index | 2 | -1.69 | 0.000 | -2.34 | -1.04 | 0% | 0.420 |
|  |  |  |  |  |  |  |  |  | Other geriatric assessment | 1 | 0.58 | 0.175 | -0.26 | 1.41 | 0% | 1.000 |
|  |  |  |  |  |  |  |  |  | Genetically modified models | 3 | -1.57 | 0.000 | -2.39 | -0.75 | 0% | 0.831 |
|  | | | | | | | | | | | | | | | | |
| **Complex V protein expression** | 12 | -0.60 | 0.230 | -1.58 | 0.38 | 87% | Species | 0.003 | Human | 4 | -1.32 | 0.004 | -2.22 | -0.41 | 67% | 0.033 |
|  |  |  |  |  |  |  |  |  | Rat | 2 | 2.30 | 0.000 | 1.13 | 3.48 | 5% | 0.305 |
|  |  |  |  |  |  |  |  |  | Mouse | 6 | -0.96 | 0.122 | -2.17 | 0.26 | 80% | 0.000 |
|  |  |  |  |  |  |  | Frailty assessment | 0.638 | Assumed based on age | 6 | -0.07 | 0.945 | -2.05 | 1.91 | 91% | 0.000 |
|  |  |  |  |  |  |  |  |  | Immobilisation/ sedentary lifestyle | 1 | -0.27 | 0.576 | -1.20 | 0.67 | 0% | 1.000 |
|  |  |  |  |  |  |  |  |  | Fried frailty index | 2 | -1.64 | 0.007 | -2.82 | -0.46 | 68% | 0.079 |
|  |  |  |  |  |  |  |  |  | Genetically modified models | 3 | -0.94 | 0.015 | -1.69 | -0.18 | 0% | 0.806 |

**Table S4 (continued): Mitochondrial respiratory chain complexes**

| ***Outcome*** | ***Primary analysis*** | | | | | | ***Heterogeneity analysis*** | | | | ***Subgroup analysis*** | | | | | |
| --- | --- | --- | --- | --- | --- | --- | --- | --- | --- | --- | --- | --- | --- | --- | --- | --- |
|  | **No. of studies** | **Estimate** | **p value** | **Lower CI** | **Upper CI** | **I^2** | **Type of heterogeneity** | **QM p value** | **No. of studies per source of heterogeneity** | | **Estimate** | **p value** | **Lower CI** | **Upper CI** | **I^2** | **Qp value** |
| **31P-MRS phospho-creatine recovery rate** | 4 | 1.69 | 0.278 | -1.37 | 4.75 | 98% | Species | NA | Human | 4 | 1.69 | 0.278 | -1.37 | 4.75 | 98% | 0.000 |
|  |  |  |  |  |  |  | Frailty assessment | 0.607 | Sarcopenia/ muscle atrophy | 3 | 2.14 | 0.321 | -2.09 | 6.37 | 98% | 0.000 |
|  |  |  |  |  |  |  |  |  | Fried frailty index | 1 | 0.46 | 0.280 | -0.38 | 1.30 | 0% | 1.000 |
|  | | | | | | | | | | | | | | | | |
| **ATP amount** | 4 | -1.69 | 0.209 | -4.33 | 0.95 | 96% | Species | 0.589 | Rat | 2 | -1.46 | 0.000 | -1.97 | -0.94 | 0% | 0.545 |
|  |  |  |  |  |  |  |  |  | Mouse | 2 | -1.95 | 0.572 | -8.71 | 4.81 | 96% | 0.000 |
|  |  |  |  |  |  |  | Frailty assessment | NA | Assumed based on age | 4 | -1.69 | 0.209 | -4.33 | 0.95 | 96% | 0.000 |
|  | | | | | | | | | | | | | | | | |
| **Citrate synthase protein expression** | 3 | -1.28 | 0.133 | -2.94 | 0.39 | 89% | Species | NA | Human | 1 | -3.10 | 0.000 | -4.41 | -1.80 | 0% | 1.000 |
|  |  |  |  |  |  |  |  |  | Rat | 1 | -0.44 | 0.106 | -0.97 | 0.09 | 0% | 1.000 |
|  |  |  |  |  |  |  |  |  | Mouse | 1 | -0.49 | 0.408 | -1.63 | 0.66 | 0% | 1.000 |
|  |  |  |  |  |  |  | Frailty assessment | 0.427 | Assumed based on age | 1 | -0.49 | 0.408 | -1.63 | 0.66 | 0% | 1.000 |
|  |  |  |  |  |  |  |  |  | Immobilisation/ sedentary lifestyle | 2 | -1.70 | 0.201 | -4.31 | 0.91 | 93% | 0.000 |
|  | | | | | | | | | | | | | | | | |
| **Citrate synthase activity** | 18 | -0.72 | 0.108 | -1.60 | 0.16 | 91% | Species | 0.156 | Human | 1 | -3.05 | 0.000 | -4.34 | -1.76 | 0% | 1.000 |
|  |  |  |  |  |  |  |  |  | Rat | 8 | -0.15 | 0.776 | -1.19 | 0.89 | 89% | 0.000 |
|  |  |  |  |  |  |  |  |  | Mouse | 9 | -1.04 | 0.147 | -2.45 | 0.37 | 91% | 0.000 |
|  |  |  |  |  |  |  | Frailty assessment | 0.430 | Assumed based on age | 15 | -0.61 | 0.245 | -1.63 | 0.42 | 91% | 0.000 |
|  |  |  |  |  |  |  |  |  | Immobilisation/ sedentary lifestyle | 2 | -1.60 | 0.249 | -4.32 | 1.12 | 93% | 0.000 |
|  |  |  |  |  |  |  |  |  | Genetically modified models | 1 | -0.62 | 0.392 | -2.04 | 0.80 | 0% | 1.000 |
|  | | | | | | | | | | | | | | | | |
| **P/O ratio** | 3 | -1.62 | 0.000 | -2.25 | -0.99 | 0% | Species | 0.074 | Human | 1 | -1.58 | 0.000 | -2.44 | -0.71 | 0% | 1.000 |
|  |  |  |  |  |  |  |  |  | Mouse | 2 | -2.17 | 0.048 | -4.31 | -0.02 | 69% | 0.071 |
|  |  |  |  |  |  |  | Frailty assessment | 0.074 | Assumed based on age | 2 | -2.17 | 0.048 | -4.31 | -0.02 | 69% | 0.071 |
|  |  |  |  |  |  |  |  |  | Fried frailty index | 1 | -1.58 | 0.000 | -2.44 | -0.71 | 0% | 1.000 |

**Table S5: Mitochondrial energy production**

**Table S6: Oxygen consumption**

| ***Outcome*** | ***Primary analysis*** | | | | | | ***Heterogeneity analysis*** | | | | ***Subgroup analysis*** | | | | | |
| --- | --- | --- | --- | --- | --- | --- | --- | --- | --- | --- | --- | --- | --- | --- | --- | --- |
|  | **No. of studies** | **Estimate** | **p value** | **Lower CI** | **Upper CI** | **I^2** | **Type of heterogeneity** | **QM p value** | **No. of studies per source of heterogeneity** | | **Estimate** | **p value** | **Lower CI** | **Upper CI** | **I^2** | **Qp value** |
| **State 3 oxygen consumption (MG)** | 10 | -1.09 | 0.001 | -1.73 | -0.44 | 70% | Species | 0.025 | Human | 3 | -1.13 | 0.000 | -1.64 | -0.62 | 0% | 0.766 |
|  |  |  |  |  |  |  |  |  | Rat | 3 | -0.58 | 0.244 | -1.56 | 0.40 | 63% | 0.075 |
|  |  |  |  |  |  |  |  |  | Mouse | 4 | -2.81 | 0.169 | -6.82 | 1.19 | 96% | 0.000 |
|  |  |  |  |  |  |  | Frailty assessment | 0.065 | Assumed based on age | 8 | -1.09 | 0.023 | -2.03 | -0.15 | 80% | 0.000 |
|  |  |  |  |  |  |  |  |  | Immobilisation/ sedentary lifestyle | 1 | -1.36 | 0.010 | -2.39 | -0.33 | 0% | 1.000 |
|  |  |  |  |  |  |  |  |  | Fried frailty index | 1 | -1.21 | 0.004 | -2.04 | -0.39 | 0% | 1.000 |
|  | | | | | | | | | | | | | | | | |
| **State 3 oxygen consumption (MP)** | 5 | -2.11 | 0.028 | -4.00 | -0.23 | 94% | Species | 0.028 | Human | 2 | -0.66 | 0.028 | -1.24 | -0.07 | 12% | 0.286 |
|  |  |  |  |  |  |  |  |  | Mouse | 3 | -3.21 | 0.035 | -6.20 | -0.22 | 94% | 0.000 |
|  |  |  |  |  |  |  | Frailty assessment | 0.470 | Assumed based on age | 2 | -4.15 | 0.074 | -8.71 | 0.41 | 92% | 0.000 |
|  |  |  |  |  |  |  |  |  | Immobilisation/ sedentary lifestyle | 1 | -0.41 | 0.255 | -1.11 | 0.29 | 0% | 1.000 |
|  |  |  |  |  |  |  |  |  | Other geriatric assessment | 1 | -1.02 | 0.022 | -1.88 | -0.15 | 0% | 1.000 |
|  |  |  |  |  |  |  |  |  | Genetically modified models | 1 | -1.59 | 0.001 | -2.55 | -0.63 | 0% | 1.000 |
|  | | | | | | | | | | | | | | | | |
| **State 4 oxygen consumption (MG)** | 8 | -0.64 | 0.001 | -1.02 | -0.25 | 15% | Species | 0.001 | Human | 2 | -0.84 | 0.004 | -1.41 | -0.27 | 0% | 0.429 |
|  |  |  |  |  |  |  |  |  | Rat | 2 | -0.02 | 0.945 | -0.70 | 0.65 | 0% | 0.571 |
|  |  |  |  |  |  |  |  |  | Mouse | 4 | -0.91 | 0.030 | -1.73 | -0.09 | 48% | 0.164 |
|  |  |  |  |  |  |  | Frailty assessment | 0.015 | Assumed based on age | 7 | -0.67 | 0.006 | -1.15 | -0.19 | 30% | 0.161 |
|  |  |  |  |  |  |  |  |  | Fried frailty index | 1 | -0.52 | 0.193 | -1.29 | 0.26 | 0% | 1.000 |
|  | | | | | | | | | | | | | | | | |
| **State 4 oxygen consumption (MP)** | 5 | -0.36 | 0.163 | -0.87 | 0.15 | 44% | Species | 0.237 | Human | 2 | -0.69 | 0.153 | -1.65 | 0.26 | 65% | 0.091 |
|  |  |  |  |  |  |  |  |  | Mouse | 3 | -0.13 | 0.683 | -0.73 | 0.48 | 27% | 0.242 |
|  |  |  |  |  |  |  | Frailty assessment | 0.607 | Assumed based on age | 2 | -0.06 | 0.909 | -1.15 | 1.02 | 65% | 0.096 |
|  |  |  |  |  |  |  |  |  | Immobilisation/ sedentary lifestyle | 1 | -0.25 | 0.485 | -0.95 | 0.45 | 0% | 1.000 |
|  |  |  |  |  |  |  |  |  | Other geriatric assessment | 1 | -1.22 | 0.007 | -2.11 | -0.33 | 0% | 1.000 |
|  |  |  |  |  |  |  |  |  | Genetically modified animal models | 1 | -0.22 | 0.614 | -1.05 | 0.62 | 0% | 1.000 |
|  | | | | | | | | | | | | | | | | |
| **VO2 max (whole body)** | 5 | -1.72 | 0.005 | -2.93 | -0.52 | 87% | Species | 0.000 | Human | 3 | -1.93 | 0.000 | -2.66 | -1.19 | 41% | 0.185 |
|  |  |  |  |  |  |  |  |  | Rat | 1 | 0.01 | 0.962 | -0.51 | 0.54 | 0% | 1.000 |
|  |  |  |  |  |  |  |  |  | Mouse | 1 | 4.31 | 0.002 | -7.02 | -1.60 | 0% | 1.000 |
|  |  |  |  |  |  |  | Frailty assessment | 0.027 | Assumed based on age | 2 | 2.60 | 0.059 | -5.30 | 0.10 | 73% | 0.053 |
|  |  |  |  |  |  |  |  |  | Immobilisation/ sedentary lifestyle | 3 | -1.38 | 0.085 | -2.94 | 0.19 | 91% | 0.000 |

| ***Outcome*** | ***Primary analysis*** | | | | | | ***Heterogeneity analysis*** | | | | ***Subgroup analysis*** | | | | | |
| --- | --- | --- | --- | --- | --- | --- | --- | --- | --- | --- | --- | --- | --- | --- | --- | --- |
|  | **No. of studies** | **Estimate** | **p value** | **Lower CI** | **Upper CI** | **I^2** | **Type of heterogeneity** | **QM p value** | **No. of studies per source of heterogeneity** | | **Estimate** | **p value** | **Lower CI** | **Upper CI** | **I^2** | **Qp value** |
| **Catalase activity** | 9 | -0.50 | 0.561 | -2.17 | 1.18 | 96% | Species | 0.958 | Human | 1 | -0.83 | 0.063 | -1.70 | 0.05 | 0% | 1.000 |
|  |  |  |  |  |  |  |  |  | Rat | 3 | -0.26 | 0.856 | -3.07 | 2.55 | 94% | 0.000 |
|  |  |  |  |  |  |  |  |  | Mouse | 5 | -0.64 | 0.665 | -3.55 | 2.27 | 98% | 0.000 |
|  |  |  |  |  |  |  | Frailty assessment | 0.856 | Assumed based on age | 7 | -0.56 | 0.625 | -2.82 | 1.70 | 97% | 0.000 |
|  |  |  |  |  |  |  |  |  | Immobilisation/ sedentary lifestyle | 2 | -0.37 | 0.376 | -1.18 | 0.45 | 54% | 0.142 |
|  | | | | | | | | | | | | | | | | |
| **Catalase gene expression** | 3 | -0.22 | 0.683 | -1.25 | 0.82 | 82% | Species | 0.666 | Human | 1 | -0.89 | 0.046 | -1.77 | -0.01 | 0% | 1.000 |
|  |  |  |  |  |  |  |  |  | Mouse | 2 | 0.09 | 0.893 | -1.25 | 1.44 | 85% | 0.009 |
|  |  |  |  |  |  |  | Frailty assessment | NA | Assumed based on age | 1 | 0.74 | 0.013 | 0.16 | 1.33 | 0% | 1.000 |
|  |  |  |  |  |  |  |  |  | Immobilisation/ sedentary lifestyle | 1 | -0.89 | 0.046 | -1.77 | -0.01 | 0% | 1.000 |
|  |  |  |  |  |  |  |  |  | Genetically modified models | 1 | -0.63 | 0.148 | -1.48 | 0.22 | 0% | 1.000 |
|  | | | | | | | | | | | | | | | | |
| **Catalase protein expression** | 6 | 0.51 | 0.112 | -0.12 | 1.14 | 67% | Species | 0.001 | Human | 2 | -0.11 | 0.683 | -0.65 | 0.42 | 0% | 0.620 |
|  |  |  |  |  |  |  |  |  | Mouse | 4 | 0.90 | 0.016 | 0.17 | 1.62 | 53% | 0.099 |
|  |  |  |  |  |  |  | Frailty assessment | 0.001 | Assumed based on age | 4 | 0.90 | 0.016 | 0.17 | 1.62 | 53% | 0.099 |
|  |  |  |  |  |  |  |  |  | Immobilisation/ sedentary lifestyle | 2 | -0.11 | 0.683 | -0.65 | 0.42 | 0% | 0.620 |
|  | | | | | | | | | | | | | | | | |
| **GPX activity** | 7 | -0.41 | 0.343 | -1.26 | 0.44 | 89% | Species | 0.068 | Rat | 3 | -1.40 | 0.090 | -3.01 | 0.22 | 82% | 0.002 |
|  |  |  |  |  |  |  |  |  | Mouse | 4 | 0.06 | 0.633 | -0.19 | 0.32 | 0% | 0.129 |
|  |  |  |  |  |  |  | Frailty assessment | 0.635 | Assumed based on age | 5 | -0.57 | 0.397 | -1.87 | 0.74 | 89% | 0.000 |
|  |  |  |  |  |  |  |  |  | Immobilisation/ sedentary lifestyle | 2 | -0.07 | 0.733 | -0.45 | 0.32 | 23% | 0.254 |
|  | | | | | | | | | | | | | | | | |
| **GPX protein expression** | 3 | -0.11 | 0.763 | -0.83 | 0.61 | 71% | Species | 0.421 | Human | 1 | -0.75 | 0.041 | -1.47 | -0.03 | 0% | 1.000 |
|  |  |  |  |  |  |  |  |  | Mouse | 2 | 0.19 | 0.658 | -0.64 | 1.01 | 58% | 0.123 |
|  |  |  |  |  |  |  | Frailty assessment | 0.265 | Assumed based on age | 1 | 0.80 | 0.155 | -0.30 | 1.88 | 0% | 1.00 |
|  |  |  |  |  |  |  |  |  | Immobilisation/ sedentary lifestyle | 2 | -0.34 | 0.268 | -0.95 | 0.27 | 60% | 0.11 |
|  | | | | | | | | | | | | | | | | |
| **HSP70 protein expression** | 5 | -0.35 | 0.763 | -1.28 | 0.59 | 68% | Species | 0.236 | Human | 1 | 0.71 | 0.169 | -0.30 | 1.72 | 0% | 1.000 |
|  |  |  |  |  |  |  |  |  | Rat | 1 | 0.47 | 0.441 | -0.73 | 1.68 | 0% | 1.000 |
|  |  |  |  |  |  |  |  |  | Mouse | 3 | -1.00 | 0.069 | -2.07 | 0.08 | 54% | 0.114 |
|  |  |  |  |  |  |  | Frailty assessment | 0.612 | Sarcopenia/ muscle atrophy | 1 | 0.71 | 0.169 | -0.30 | 1.72 | 0% | 1.000 |
|  |  |  |  |  |  |  |  |  | Assumed based on age | 2 | -0.34 | 0.685 | -1.95 | 1.28 | 70% | 0.066 |
|  |  |  |  |  |  |  |  |  | Genetically modified models | 2 | -1.05 | 0.275 | -2.95 | 0.84 | 74% | 0.051 |

**Table S7: Oxidative stress**

**Continued on the next page**

**Table S7 (continued): Oxidative stress**

| ***Outcome*** | ***Primary analysis*** | | | | | | ***Heterogeneity analysis*** | | | | ***Subgroup analysis*** | | | | | |
| --- | --- | --- | --- | --- | --- | --- | --- | --- | --- | --- | --- | --- | --- | --- | --- | --- |
|  | **No. of studies** | **Estimate** | **p value** | **Lower CI** | **Upper CI** | **I^2** | **Type of heterogeneity** | **QM p value** | **No. of studies per source of heterogeneity** | | **Estimate** | **p value** | **Lower CI** | **Upper CI** | **I^2** | **Qp value** |
| **mtROS production** | 3 | 0.88 | 0.026 | 0.11 | 1.66 | 47% | Species | NA | Human | 1 | 0.42 | 0.286 | -0.35 | 1.19 | 0% | 1.000 |
|  |  |  |  |  |  |  |  |  | Rat | 1 | 0.74 | 0.111 | -0.17 | 1.64 | 0% | 1.000 |
|  |  |  |  |  |  |  |  |  | Mouse | 1 | 2.02 | 0.004 | 0.63 | 3.41 | 0% | 1.000 |
|  |  |  |  |  |  |  | Frailty assessment | NA | Assumed based on age | 1 | 0.74 | 0.111 | -0.17 | 1.64 | 0% | 1.000 |
|  |  |  |  |  |  |  |  |  | Fried frailty index | 1 | 0.42 | 0.286 | -0.35 | 1.19 | 0% | 1.000 |
|  |  |  |  |  |  |  |  |  | Genetically modified models | 1 | 2.02 | 0.004 | 0.63 | 3.41 | 0% | 1.000 |
|  | | | | | | | | | | | | | | | | |
| **ROS in respiration (state 2)** | 4 | 0.49 | 0.323 | -0.49 | 1.48 | 75% | Species | 0.020 | Human | 2 | -0.16 | 0.560 | -0.68 | 0.37 | 0% | 0.838 |
|  |  |  |  |  |  |  |  |  | Rat | 1 | 0.66 | 0.261 | -0.49 | 1.80 | 0% | 1.000 |
|  |  |  |  |  |  |  |  |  | Mouse | 1 | 2.37 | 0.004 | 0.75 | 3.98 | 0% | 1.000 |
|  |  |  |  |  |  |  | Frailty assessment | 0.038 | Assumed based on age | 2 | 0.17 | 0.641 | -0.53 | 0.86 | 9% | 0.293 |
|  |  |  |  |  |  |  |  |  | Immobilisation/ sedentary lifestyle | 1 | -0.20 | 0.568 | -0.90 | 0.49 | 0% | 1.000 |
|  |  |  |  |  |  |  |  |  | Genetically modified models | 1 | 2.37 | 0.004 | 0.75 | 3.98 | 0% | 1.000 |
|  | | | | | | | | | | | | | | | | |
| **ROS in respiration (state 3)** | 5 | 0.25 | 0.509 | -0.49 | 0.98 | 69% | Species | 0.008 | Human | 2 | -0.52 | 0.056 | -1.06 | 0.01 | 0% | 0.333 |
|  |  |  |  |  |  |  |  |  | Rat | 2 | 1.09 | 0.007 | 0.30 | 1.88 | 0% | 0.610 |
|  |  |  |  |  |  |  |  |  | Mouse | 1 | 0.40 | 0.371 | -0.48 | 1.29 | 0% | 1.000 |
|  |  |  |  |  |  |  | Frailty assessment | 0.136 | Assumed based on age | 4 | 0.51 | 0.129 | -0.15 | 1.17 | 46% | 0.136 |
|  |  |  |  |  |  |  |  |  | Immobilisation/ sedentary lifestyle | 1 | -0.76 | 0.039 | -1.48 | -0.04 | 0% | 1.000 |
|  | | | | | | | | | | | | | | | | |
| **ROS in respiration (state 4)** | 7 | 1.17 | 0.121 | -0.31 | 2.66 | 94% | Species | 0.460 | Human | 2 | 0.11 | 0.676 | -0.41 | 0.64 | 0% | 0.473 |
|  |  |  |  |  |  |  |  |  | Rat | 1 | 1.72 | 0.011 | 0.40 | 3.05 | 0% | 1.000 |
|  |  |  |  |  |  |  |  |  | Mouse | 4 | 1.63 | 0.215 | -0.95 | 4.22 | 96% | 0.000 |
|  |  |  |  |  |  |  | Frailty assessment | 0.542 | Assumed based on age | 4 | 1.63 | 0.130 | -0.48 | 3.73 | 94% | 0.000 |
|  |  |  |  |  |  |  |  |  | Immobilisation/ sedentary lifestyle | 1 | 0.28 | 0.431 | -0.42 | 0.98 | 0% | 1.000 |
|  |  |  |  |  |  |  |  |  | Genetically modified models | 2 | 0.78 | 0.705 | -3.26 | 4.82 | 95% | 0.000 |

**Continued on the next page**

| ***Outcome*** | ***Primary analysis*** | | | | | | ***Heterogeneity analysis*** | | | | ***Subgroup analysis*** | | | | | |
| --- | --- | --- | --- | --- | --- | --- | --- | --- | --- | --- | --- | --- | --- | --- | --- | --- |
|  | **No. of studies** | **Estimate** | **p value** | **Lower CI** | **Upper CI** | **I^2** | **Type of heterogeneity** | **QM p value** | **No. of studies per source of heterogeneity** | | **Estimate** | **p value** | **Lower CI** | **Upper CI** | **I^2** | **Qp value** |
| **SOD2 activity** | 11 | -0.17 | 0.801 | -1.47 | 1.14 | 95% | Species | 0.543 | Human | 2 | -1.01 | 0.381 | -3.28 | 1.26 | 92% | 0.001 |
|  |  |  |  |  |  |  |  |  | Rat | 3 | -1.26 | 0.028 | -2.39 | -0.14 | 57% | 0.104 |
|  |  |  |  |  |  |  |  |  | Mouse | 6 | 0.68 | 0.516 | -1.37 | 2.73 | 97% | 0.000 |
|  |  |  |  |  |  |  | Frailty assessment | 0.598 | Assumed based on age | 7 | -0.41 | 0.347 | -1.27 | 0.45 | 79% | 0.001 |
|  |  |  |  |  |  |  |  |  | Immobilisation/ sedentary lifestyle | 3 | 1.06 | 0.628 | -3.24 | 5.37 | 99% | 0.000 |
|  |  |  |  |  |  |  |  |  | Genetically modified models | 1 | -2.06 | 0.004 | -3.46 | -0.66 | 0% | 1.000 |
|  | | | | | | | | | | | | | | | | |
| **SOD activity (total SOD)** | 4 | -0.53 | 0.678 | -3.04 | 1.98 | 95% | Species | 0.763 | Human | 1 | -3.30 | 0.000 | -4.65 | -1.95 | 0% | 1.000 |
|  |  |  |  |  |  |  |  |  | Rat | 2 | 0.63 | 0.780 | -3.78 | 5.03 | 95% | 0.000 |
|  |  |  |  |  |  |  |  |  | Mouse | 1 | -0.06 | 0.874 | -0.75 | 0.64 | 0% | 1.000 |
|  |  |  |  |  |  |  | Frailty assessment | 0.666 | Assumed based on age | 2 | 0.63 | 0.780 | -3.78 | 5.03 | 95% | 0.000 |
|  |  |  |  |  |  |  |  |  | Immobilisation/ sedentary lifestyle | 2 | -1.63 | 0.316 | -4.80 | 1.55 | 94% | 0.000 |
|  |  |  |  |  |  |  |  |  |  |  |  |  |  |  |  |  |
| **SOD1 gene expression** | 3 | 1.08 | 0.463 | -1.80 | 3.96 | 93% | Species | 0.671 | Human | 1 | -0.47 | 0.282 | -1.32 | 0.38 | 0% | 1.000 |
|  |  |  |  |  |  |  |  |  | Mouse | 2 | 1.96 | 0.378 | -2.40 | 6.32 | 92% | 0.000 |
|  |  |  |  |  |  |  | Frailty assessment | NA | Assumed based on age | 1 | -0.19 | 0.765 | -1.43 | 1.05 | 0% | 1.000 |
|  |  |  |  |  |  |  |  |  | Immobilisation/ sedentary lifestyle | 1 | -0.47 | 0.282 | -1.32 | 0.38 | 0% | 1.000 |
|  |  |  |  |  |  |  |  |  | Genetically modified models | 1 | 4.26 | 0.000 | 2.22 | 6.31 | 0% | 1.000 |
|  | | | | | | | | | | | | | | | | |
| **SOD1 protein expression** | 5 | 1.41 | 0.022 | 0.20 | 2.61 | 83% | Species | 0.000 | Human | 2 | 0.14 | 0.618 | -0.40 | 0.67 | 0% | 0.400 |
|  |  |  |  |  |  |  |  |  | Rat | 1 | 2.79 | 0.000 | 1.56 | 4.02 | 0% | 1.000 |
|  |  |  |  |  |  |  |  |  | Mouse | 2 | 2.16 | 0.000 | 0.99 | 3.34 | 0% | 0.368 |
|  |  |  |  |  |  |  | Frailty assessment | 0.000 | Assumed based on age | 3 | 2.46 | 0.000 | 1.61 | 3.31 | 0% | 0.513 |
|  |  |  |  |  |  |  |  |  | Immobilisation/ sedentary lifestyle | 2 | 0.14 | 0.618 | -0.40 | 0.67 | 0% | 0.400 |
|  | | | | | | | | | | | | | | | | |
| **SOD2 gene expression** | 6 | -0.61 | 0.020 | -1.12 | -0.09 | 65% | Species | 0.039 | Human | 1 | -0.97 | 0.033 | -1.85 | -0.08 | 0% | 1.000 |
|  |  |  |  |  |  |  |  |  | Rat | 1 | -1.39 | 0.015 | -2.51 | -0.27 | 0% | 1.000 |
|  |  |  |  |  |  |  |  |  | Mouse | 4 | -0.38 | 0.168 | -0.91 | 0.16 | 60% | 0.056 |
|  |  |  |  |  |  |  | Frailty assessment | 0.219 | Assumed based on age | 2 | -0.63 | 0.514 | -2.52 | 1.26 | 83% | 0.015 |
|  |  |  |  |  |  |  |  |  | Immobilisation/ sedentary lifestyle | 3 | -0.74 | 0.011 | -1.31 | -0.17 | 46% | 0.165 |
|  |  |  |  |  |  |  |  |  | Genetically modified models | 1 | -0.48 | 0.269 | -1.33 | 0.37 | 0% | 1.000 |
|  | | | | | | | | | | | | | | | | |
| **SOD2 protein expression** | 9 | -0.86 | 0.026 | -1.61 | -0.10 | 78% | Species | 0.003 | Human | 4 | -1.17 | 0.000 | -1.65 | -0.68 | 17% | 0.276 |
|  |  |  |  |  |  |  |  |  | Rat | 1 | -2.31 | 0.000 | -3.44 | -1.17 | 0% | 1.000 |
|  |  |  |  |  |  |  |  |  | Mouse | 4 | 0.10 | 0.900 | -1.44 | 1.63 | 81% | 0.007 |
|  |  |  |  |  |  |  | Frailty assessment | 0.069 | Assumed based on age | 5 | -0.39 | 0.621 | -1.91 | 1.14 | 86% | 0.000 |
|  |  |  |  |  |  |  |  |  | Immobilisation/ sedentary lifestyle | 4 | -1.17 | 0.000 | -1.65 | -0.68 | 17% | 0.276 |

**Table S7 (continued): Oxidative stress**

**Table S8: Apoptosis and autophagy**

| ***Outcome*** | ***Primary analysis*** | | | | | | ***Heterogeneity analysis*** | | | | ***Subgroup analysis*** | | | | | |
| --- | --- | --- | --- | --- | --- | --- | --- | --- | --- | --- | --- | --- | --- | --- | --- | --- |
|  | **No. of studies** | **Estimate** | **p value** | **Lower CI** | **Upper CI** | **I^2** | **Type of heterogeneity** | **MQ p value** | **No. of studies per source of heterogeneity** | | **Estimate** | **p value** | **Lower CI** | **Upper CI** | **I^2** | **Qp value** |
| **AIF release** | 3 | 1.45 | 0.001 | 0.61 | 2.30 | 40% | Species | 0.000 | Rat | 2 | 1.78 | 0.000 | 0.86 | 2.71 | 27% | 0.241 |
|  |  |  |  |  |  |  |  |  | Mouse | 1 | 0.72 | 0.229 | -0.45 | 1.88 | 0% | 1.000 |
|  |  |  |  |  |  |  | Frailty assessment | 0.000 | Assumed based on age | 2 | 1.78 | 0.000 | 0.86 | 2.71 | 27% | 0.241 |
|  |  |  |  |  |  |  |  |  | Genetically modified models | 1 | 0.72 | 0.229 | -0.45 | 1.88 | 0% | 1.000 |
|  | | | | | | | | | | | | | | | | |
| **BAX protein expression** | 7 | -0.63 | 0.385 | -2.06 | 0.79 | 91% | Species | 0.633 | Human | 1 | 0.30 | 0.529 | -0.63 | 1.22 | 0% | 1.000 |
|  |  |  |  |  |  |  |  |  | Rat | 7 | -0.82 | 0.345 | -2.51 | 0.87 | 91% | 0.000 |
|  |  |  |  |  |  |  | Frailty assessment | 0.844 | Assumed based on age | 5 | -0.72 | 0.499 | -2.81 | 1.38 | 92% | 0.000 |
|  |  |  |  |  |  |  |  |  | Immobilisation/ sedentary lifestyle | 1 | -1.40 | 0.015 | -2.52 | -0.27 | 0% | 1.000 |
|  |  |  |  |  |  |  |  |  | Other geriatric assessment | 1 | 0.30 | 0.529 | -0.63 | 1.22 | 0% | 1.000 |
|  | | | | | | | | | | | | | | | | |
| **Bcl-2 protein expression** | 6 | -0.57 | 0.563 | -2.51 | 1.37 | 93% | Species | 0.343 | Rat | 5 | -0.03 | 0.979 | -1.99 | 1.94 | 92% | 0.000 |
|  |  |  |  |  |  |  |  |  | Mouse | 1 | -3.29 | 0.000 | -4.64 | -1.95 | 0% | 1.000 |
|  |  |  |  |  |  |  | Frailty assessment | 0.000 | Assumed based on age | 4 | 0.84 | 0.837 | -0.18 | 1.86 | 68% | 0.021 |
|  |  |  |  |  |  |  |  |  | Immobilisation/ sedentary lifestyle | 1 | -3.94 | 0.000 | -5.64 | -2.24 | 0% | 1.000 |
|  |  |  |  |  |  |  |  |  | Other geriatric assessment | 1 | -3.29 | 0.000 | -4.63 | -1.94 | 0% | 1.000 |
|  | | | | | | | | | | | | | | | | |
| **Beclin1 protein expression** | 6 | -0.67 | 0.703 | -4.09 | 2.75 | 97% | Species | 0.579 | Human | 1 | -0.31 | 0.571 | -1.36 | 0.75 | 0% | 1.000 |
|  |  |  |  |  |  |  |  |  | Dog | 1 | 1.58 | 0.003 | 0.54 | 2.62 | 0% | 1.000 |
|  |  |  |  |  |  |  |  |  | Rat | 1 | -7.56 | 0.000 | -10.37 | -4.75 | 0% | 1.000 |
|  |  |  |  |  |  |  |  |  | Mouse | 3 | 0.59 | 0.819 | -4.46 | 5.65 | 97% | 0.000 |
|  |  |  |  |  |  |  | Frailty assessment | 0.285 | Sarcopenia/ muscle atrophy | 1 | 1.58 | 0.003 | 0.54 | 2.62 | 0% | 1.000 |
|  |  |  |  |  |  |  |  |  | Assumed based on age | 2 | 2.70 | 0.296 | -2.36 | 7.77 | 94% | 0.000 |
|  |  |  |  |  |  |  |  |  | Immobilisation/ sedentary lifestyle | 1 | -7.56 | 0.000 | -10.37 | -4.75 | 0% | 1.000 |
|  |  |  |  |  |  |  |  |  | Other geriatric assessment | 1 | -0.301 | 0.571 | -1.36 | 0.75 | 0% | 1.000 |
|  |  |  |  |  |  |  |  |  | Genetically modified models | 1 | -3.62 | 0.000 | -5.05 | -2.20 | 0% | 1.000 |
|  | | | | | | | | | | | | | | | | |
| **Caspase 3 activity** | 9 | 0.58 | 0.084 | -0.08 | 1.24 | 75% | Species | 0.000 | Rat | 6 | 1.79 | 0.003 | 0.61 | 2.97 | 75% | 0.028 |
|  |  |  |  |  |  |  |  |  | Mouse | 3 | 0.00 | 0.988 | -0.41 | 0.41 | 0% | 0.655 |
|  |  |  |  |  |  |  | Frailty assessment | 0.000 | Assumed based on age | 7 | 0.21 | 0.364 | -0.25 | 0.67 | 41% | 0.126 |
|  |  |  |  |  |  |  |  |  | Genetically modified models | 2 | 2.35 | 0.001 | 0.99 | 3.71 | 50% | 0.158 |
|  | | | | | | | | | | | | | | | | |
| **Caspase 3 protein expression** | 5 | 0.66 | 0.527 | -1.39 | 2.72 | 92% | Species | 0.868 | Human | 1 | 0.79 | 0.105 | -0.16 | 1.74 | 0% | 1.000 |
|  |  |  |  |  |  |  |  |  | Rat | 4 | 0.62 | 0.656 | -2.10 | 3.34 | 94% | 0.000 |
|  |  |  |  |  |  |  | Frailty assessment | 0.979 | Assumed based on age | 3 | 0.72 | 0.716 | -3.18 | 4.63 | 94% | 0.000 |
|  |  |  |  |  |  |  |  |  | Immobilisation/ sedentary lifestyle | 1 | 0.26 | 0.616 | -0.76 | 1.28 | 0% | 1.000 |
|  |  |  |  |  |  |  |  |  | Other geriatric assessment | 1 | 0.79 | 0.105 | -0.16 | 1.74 | 0% | 1.000 |

**Continued on the next page**

| ***Outcome*** | ***Primary analysis*** | | | | | | ***Heterogeneity analysis*** | | | | ***Subgroup analysis*** | | | | | |
| --- | --- | --- | --- | --- | --- | --- | --- | --- | --- | --- | --- | --- | --- | --- | --- | --- |
|  | **No. of studies** | **Estimate** | **p value** | **Lower CI** | **Upper CI** | **I^2** | **Type of heterogeneity** | **MQ p value** | **No. of studies per source of heterogeneity** | | **Estimate** | **p value** | **Lower CI** | **Upper CI** | **I^2** | **Qp value** |
| **Caspase 9 activity** | 4 | 0.52 | 0.195 | -0.26 | 1.30 | 72% | Species | 0.002 | Rat | 2 | -0.16 | 0.627 | -0.81 | 0.49 | 0% | 0.974 |
|  |  |  |  |  |  |  |  |  | Mouse | 2 | 1.09 | 0.008 | 0.29 | 1.89 | 53% | 0.145 |
|  |  |  |  |  |  |  | Frailty assessment | 0.040 | Assumed based on age | 1 | 1.59 | 0.001 | 0.63 | 2.55 | 0% | 1.000 |
|  |  |  |  |  |  |  |  |  | Genetically modified models | 3 | 0.23 | 0.498 | -0.44 | 0.90 | 52% | 0.122 |
|  | | | | | | | | | | | | | | | | |
| **Caspase 9 protein expression** | 3 | -2.74 | 0.310 | -8.04 | 2.55 | 98% | Species | NA | Rat | 3 | -2.74 | 0.310 | -8.04 | 2.55 | 98% | 0.000 |
|  |  |  |  |  |  |  | Frailty assessment | NA | Assumed based on age | 3 | -2.74 | 0.310 | -8.04 | 2.55 | 98% | 0.000 |
|  | | | | | | | | | | | | | | | | |
| **Cytochrome c release** | 5 | 0.50 | 0.211 | -0.28 | 1.29 | 64% | Species | 0.848 | Human | 1 | 0.37 | 0.433 | -0.56 | 1.30 | 0% | 1.000 |
|  |  |  |  |  |  |  |  |  | Rat | 3 | 0.48 | 0.518 | -0.97 | 1.92 | 81% | 0.005 |
|  |  |  |  |  |  |  |  |  | Mouse | 1 | 0.71 | 0.234 | -0.46 | 1.88 | 0% | 1.000 |
|  |  |  |  |  |  |  | Frailty assessment | 0.848 | Assumed based on age | 4 | 0.45 | 0.518 | -0.97 | 1.92 | 81% | 0.005 |
|  |  |  |  |  |  |  |  |  | Other geriatric assessment | 1 | 0.37 | 0.433 | -0.56 | 1.30 | 0% | 1.000 |
|  |  |  |  |  |  |  |  |  | Genetically modified models | 1 | 0.71 | 0.234 | -0.46 | 1.88 | 0% | 1.000 |
|  | | | | | | | | | | | | | | | | |
| **LC3 protein expression** | 8 | -0.17 | 0.665 | -0.94 | 0.60 | 76% | Species | 0.299 | Human | 1 | -0.81 | 0.058 | -1.64 | 0.03 | 0% | 1.000 |
|  |  |  |  |  |  |  |  |  | Dog | 1 | 1.28 | 0.013 | 0.27 | 2.29 | 0% | 1.000 |
|  |  |  |  |  |  |  |  |  | Rat | 2 | -1.08 | 0.005 | -1.83 | -0.33 | 0% | 0.571 |
|  |  |  |  |  |  |  |  |  | Mouse | 4 | 0.11 | 0.850 | -1.04 | 1.27 | 75% | 0.008 |
|  |  |  |  |  |  |  | Frailty assessment | 0.803 | Sarcopenia/ muscle atrophy | 2 | 0.22 | 0.836 | -1.83 | 2.26 | 90% | 0.002 |
|  |  |  |  |  |  |  |  |  | Assumed based on age | 4 | 0.12 | 0.843 | -1.05 | 1.28 | 74% | 0.010 |
|  |  |  |  |  |  |  |  |  | Immobilisation/ sedentary lifestyle | 1 | -1.31 | 0.020 | -2.42 | -0.20 | 0% | 1.000 |
|  |  |  |  |  |  |  |  |  | Genetically modified models | 1 | -0.85 | 0.068 | -1.77 | 0.06 | 0% | 1.000 |
|  | | | | | | | | | | | | | | | | |
| **Parkin protein expression** | 3 | 0.88 | 0.294 | -0.76 | 2.53 | 82% | Species | 0.001 | Human | 1 | -0.67 | 0.220 | -1.75 | 0.40 | 0% | 1.000 |
|  |  |  |  |  |  |  |  |  | Mouse | 2 | 1.72 | 0.000 | 0.80 | 2.64 | 0% | 0.438 |
|  |  |  |  |  |  |  | Frailty assessment | 0.001 | Assumed based on age | 2 | 1.72 | 0.000 | 0.80 | 2.64 | 0% | 0.438 |
|  |  |  |  |  |  |  |  |  | Other geriatric assessment | 1 | -0.67 | 0.220 | -1.75 | 0.40 | 0% | 1.000 |
|  | | | | | | | | | | | | | | | | |
| **XIAP protein expression** | 3 | 1.08 | 0.126 | -0.30 | 2.45 | 81% | Species | 0.389 | Rat | 2 | 0.77 | 0.462 | -1.28 | 2.82 | 88% | 0.003 |
|  |  |  |  |  |  |  |  |  | Mouse | 1 | 1.75 | 0.003 | 0.60 | 2.91 | 0% | 1.000 |
|  |  |  |  |  |  |  | Frailty assessment | NA | Assumed based on age | 3 | 1.08 | 0.126 | -0.30 | 2.45 | 81% | 0.003 |

**Table S8 (continued): Apoptosis and autophagy**

| ***Outcome*** | ***Primary analysis*** | | | | | | ***Heterogeneity analysis*** | | | | ***Subgroup analysis*** | | | | | |
| --- | --- | --- | --- | --- | --- | --- | --- | --- | --- | --- | --- | --- | --- | --- | --- | --- |
|  | **No. of studies** | **Estimate** | **p value** | **Lower CI** | **Upper CI** | **I^2** | **Type of heterogeneity** | **QM p value** | **No. of studies per source of heterogeneity** | | **Estimate** | **p value** | **Lower CI** | **Upper CI** | **I^2** | **Qp value** |
| **AMPK protein expression** | 6 | -1.14 | 0.205 | -2.90 | 0.62 | 91% | Species | 0.776 | Human | 1 | 1.12 | 0.051 | -0.01 | 2.25 | 0% | 1.000 |
|  |  |  |  |  |  |  |  |  | Monkey | 1 | -1.26 | 0.159 | -3.01 | 0.49 | 0% | 1.000 |
|  |  |  |  |  |  |  |  |  | Rat | 2 | -2.12 | 0.375 | -6.81 | 2.57 | 95% | 0.000 |
|  |  |  |  |  |  |  |  |  | Mouse | 2 | -1.41 | 0.424 | -4.88 | 2.05 | 89% | 0.002 |
|  |  |  |  |  |  |  | Frailty assessment | 0.000 | Assumed based on age | 3 | 0.04 | 0.910 | -0.58 | 0.65 | 0% | 0.301 |
|  |  |  |  |  |  |  |  |  | Immobilisation/ sedentary lifestyle | 1 | -4.60 | 0.000 | -6.49 | -2.71 | 0% | 1.000 |
|  |  |  |  |  |  |  |  |  | Other geriatric assessment | 1 | 1.12 | 0.051 | -0.01 | 2.25 | 0% | 1.000 |
|  |  |  |  |  |  |  |  |  | Genetically modified models | 1 | -3.25 | 0.001 | -5.15 | -1.36 | 0% | 1.000 |
|  | | | | | | | | | | | | | | | | |
| **NRF-1 gene expression** | 5 | -1.99 | 0.000 | -1.09 | 2.08 | 57% | Species | 0.000 | Human | 1 | -0.88 | 0.116 | -1.98 | 0.22 | 0% | 1.000 |
|  |  |  |  |  |  |  |  |  | Rat | 1 | -1.18 | 0.059 | -2.41 | 0.04 | 0% | 1.000 |
|  |  |  |  |  |  |  |  |  | Mouse | 3 | -2.95 | 0.000 | -4.08 | -1.83 | 0% | 0.455 |
|  |  |  |  |  |  |  | Frailty assessment | 0.007 | Assumed based on age | 2 | -2.55 | 0.119 | -5.75 | 0.65 | 75% | 0.045 |
|  |  |  |  |  |  |  |  |  | Other geriatric assessment | 1 | -0.88 | 0.116 | -1.98 | 0.22 | 0% | 1.000 |
|  |  |  |  |  |  |  |  |  | Genetically modified models | 2 | -2.70 | 0.000 | -3.92 | -1.48 | 0% | 0.533 |
|  | | | | | | | | | | | | | | | | |
| **NRF1 protein expression** | 4 | -1.42 | 0.284 | -4.02 | 1.18 | 97% | Species | 0.910 | Human | 1 | -0.21 | 0.622 | -1.03 | 0.61 | 0% | 1.000 |
|  |  |  |  |  |  |  |  |  | Rat | 1 | -1.59 | 0.000 | -2.45 | -0.73 | 0% | 1.000 |
|  |  |  |  |  |  |  |  |  | Mouse | 2 | -2.01 | 0.523 | -8.16 | 4.15 | 98% | 0.000 |
|  |  |  |  |  |  |  | Frailty assessment | NA | Assumed based on age | 1 | -1.59 | 0.000 | -2.45 | -0.73 | 0% | 1.000 |
|  |  |  |  |  |  |  |  |  | Immobilisation/ sedentary lifestyle | 1 | -5.17 | 0.000 | -6.62 | -3.73 | 0% | 1.000 |
|  |  |  |  |  |  |  |  |  | Other geriatric assessment | 1 | -0.21 | 0.622 | -1.03 | 0.61 | 0% | 1.000 |
|  |  |  |  |  |  |  |  |  | Genetically modified models | 1 | 1.11 | 0.021 | 0.17 | 2.05 | 0% | 1.000 |
|  | | | | | | | | | | | | | | | | |
| **IGF-1 (in serum)** | 4 | -3.87 | 0.174 | -9.44 | 1.71 | 99% | Species | 0.575 | Human | 2 | -4.83 | 0.344 | -14.83 | 5.17 | 99% | 0.000 |
|  |  |  |  |  |  |  |  |  | Rat | 1 | -7.48 | 0.000 | -9.96 | -5.00 | 0% | 1.000 |
|  |  |  |  |  |  |  |  |  | Mouse | 1 | 1.54 | 0.002 | 0.54 | 2.54 | 0% | 1.000 |
|  |  |  |  |  |  |  | Frailty assessment | NA | Sarcopenia/ muscle atrophy | 1 | 0.27 | 0.336 | -0.28 | 0.82 | 0% | 1.000 |
|  |  |  |  |  |  |  |  |  | Assumed based on age | 1 | -7.48 | 0.000 | -9.96 | -5.00 | 0% | 1.000 |
|  |  |  |  |  |  |  |  |  | Fried frailty index | 1 | -9.94 | 0.000 | -10.69 | -9.19 | 0% | 1.000 |
|  |  |  |  |  |  |  |  |  | Genetically modified models | 1 | 1.54 | 0.002 | 0.54 | 2.54 | 0% | 1.000 |

**Table S9: Regulators of mitochondrial function**

**Continued on the next page**

| ***Outcome*** | ***Primary analysis*** | | | | | | ***Heterogeneity analysis*** | | | | ***Subgroup analysis*** | | | | | |
| --- | --- | --- | --- | --- | --- | --- | --- | --- | --- | --- | --- | --- | --- | --- | --- | --- |
|  | **No. of studies** | **Estimate** | **p value** | **Lower CI** | **Upper CI** | **I^2** | **Type of heterogeneity** | **QM p value** | **No. of studies per source of heterogeneity** | | **Estimate** | **p value** | **Lower CI** | **Upper CI** | **I^2** | **Qp value** |
| **PGC-1 protein expression** | 15 | -1.23 | 0.113 | -2.76 | 0.29 | 97% | Species | 0.463 | Human | 2 | -0.37 | 0.206 | -0.95 | 0.21 | 0% | 0.570 |
|  |  |  |  |  |  |  |  |  | Monkey | 1 | -0.55 | 0.506 | -2.18 | 1.08 | 0% | 1.000 |
|  |  |  |  |  |  |  |  |  | Rat | 5 | -2.80 | 0.086 | -5.99 | 0.40 | 98% | 0.000 |
|  |  |  |  |  |  |  |  |  | Mouse | 7 | -0.56 | 0.673 | -3.15 | 2.03 | 97% | 0.000 |
|  |  |  |  |  |  |  | Frailty assessment | 0.744 | Sarcopenia/ muscle atrophy | 1 | -0.21 | 0.606 | -1.01 | 0.59 | 0% | 1.000 |
|  |  |  |  |  |  |  |  |  | Assumed based on age | 5 | -0.40 | 0.752 | -2.88 | 2.08 | 94% | 0.000 |
|  |  |  |  |  |  |  |  |  | Immobilisation/ sedentary lifestyle | 4 | -2.28 | 0.384 | -7.42 | 2.86 | 99% | 0.000 |
|  |  |  |  |  |  |  |  |  | Other geriatric assessment | 1 | -0.55 | 0.198 | -1.38 | 0.29 | 0% | 1.000 |
|  |  |  |  |  |  |  |  |  | Genetically modified models | 4 | -2.11 | 0.149 | -4.97 | 0.76 | 94% | 0.000 |
|  | | | | | | | | | | | | | | | | |
| **PGC1 gene expression** | 11 | -2.98 | 0.053 | -5.99 | 0.03 | 97% | Species | 0.004 | Rat | 1 | -15.00 | 0.000 | -21.09 | -8.89 | 0% | 1.000 |
|  |  |  |  |  |  |  |  |  | Mouse | 10 | -2.04 | 0.119 | -4.60 | 0.52 | 95% | 0.000 |
|  |  |  |  |  |  |  | Frailty assessment | 0.081 | Assumed based on age | 5 | -5.10 | 0.052 | -10.23 | 0.04 | 97% | 0.000 |
|  |  |  |  |  |  |  |  |  | Genetically modified models | 6 | -1.42 | 0.450 | -5.10 | 2.26 | 97% | 0.000 |
|  | | | | | | | | | | | | | | | | |
| **SIRT3 protein expression** | 4 | -1.45 | 0.001 | -2.33 | -0.58 | 59% | Species | 0.000 | Human | 2 | -0.87 | 0.005 | -1.49 | -0.26 | 0% | 0.625 |
|  |  |  |  |  |  |  |  |  | Rat | 1 | -2.84 | 0.000 | -4.25 | -1.43 | 0% | 1.000 |
|  |  |  |  |  |  |  |  |  | Mouse | 1 | -1.78 | 0.017 | -3.25 | -0.32 | 0% | 1.000 |
|  |  |  |  |  |  |  | Frailty assessment | 0.095 | Immobilisation/ sedentary lifestyle | 2 | -1.85 | 0.039 | -3.61 | -0.09 | 78% | 0.035 |
|  |  |  |  |  |  |  |  |  | Other geriatric assessment | 1 | -0.73 | 0.091 | -1.57 | 0.12 | 0% | 1.000 |
|  |  |  |  |  |  |  |  |  | Genetically modified models | 1 | -1.78 | 0.017 | -3.25 | -0.32 | 0% | 1.000 |
|  | | | | | | | | | | | | | | | | |
| **TFAM gene expression** | 5 | -2.42 | 0.010 | -4.25 | -0.58 | 86% | Species | NA | Mouse | 5 | -2.42 | 0.01 | -4.25 | -0.58 | 86% | 0.002 |
|  |  |  |  |  |  |  | Frailty assessment | 0.074 | Assumed based on age | 2 | -1.75 | 0.041 | -3.4 | -0.07 | 49% | 0.162 |
|  |  |  |  |  |  |  |  |  | Genetically modified models | 3 | -3.07 | 0.084 | -6.56 | 0.42 | 93% | 0.000 |
|  | | | | | | | | | | | | | | | | |
| **TFAM protein expression** | 6 | -1.06 | 0.409 | -3.57 | 1.45 | 97% | Species | 0.793 | Human | 1 | -0.24 | 0.563 | -1.06 | 0.58 | 0% | 1.000 |
|  |  |  |  |  |  |  |  |  | Rat | 1 | 1.11 | 0.021 | 0.17 | 2.05 | 0% | 1.000 |
|  |  |  |  |  |  |  |  |  | Mouse | 4 | -1.86 | 0.331 | -5.60 | 1.89 | 98% | 0.000 |
|  |  |  |  |  |  |  | Frailty assessment | 0.000 | Assumed based on age | 2 | 0.72 | 0.155 | -0.27 | 1.71 | 33% | 0.223 |
|  |  |  |  |  |  |  |  |  | Immobilisation/ sedentary lifestyle | 1 | -7.72 | 0.000 | -9.73 | -5.70 | 0% | 1.000 |
|  |  |  |  |  |  |  |  |  | Other geriatric assessment | 1 | -0.24 | 0.563 | -1.06 | 0.58 | 0% | 1.000 |
|  |  |  |  |  |  |  |  |  | Genetically modified models | 2 | -0.06 | 0.942 | -1.72 | 1.60 | 88% | 0.004 |

**Table S9 (continued): Regulators of mitochondrial function**

**Table S10: Measures of iron metabolism**

| ***Outcome*** | ***Primary analysis*** | | | | | | ***Heterogeneity analysis*** | | | | ***Subgroup analysis*** | | | | | |
| --- | --- | --- | --- | --- | --- | --- | --- | --- | --- | --- | --- | --- | --- | --- | --- | --- |
|  | **No. of studies** | **Estimate** | **p value** | **Lower CI** | **Upper CI** | **I^2** | **Type of heterogeneity** | **QM p value** | **No. of studies per source of heterogeneity** | | **Estimate** | **p value** | **Lower CI** | **Upper CI** | **I^2** | **Qp value** |
| **Haemoglobin** | 11 | -1.51 | 0.006 | -2.59 | -0.43 | 98% | Species | 0.001 | Human | 6 | -0.65 | 0.001 | -1.03 | -0.27 | 86% | 0.003 |
|  |  |  |  |  |  |  |  |  | Mouse | 5 | -2.76 | 0.016 | -5.02 | -0.51 | 94% | 0.000 |
|  |  |  |  |  |  |  | Frailty assessment | 0.000 | Sarcopenia/ muscle atrophy | 1 | 0.43 | 0.294 | -0.37 | 1.24 | 0% | 1.000 |
|  |  |  |  |  |  |  |  |  | Assumed based on age | 3 | -1.00 | 0.008 | -1.75 | -0.26 | 42% | 0.185 |
|  |  |  |  |  |  |  |  |  | Fried frailty index | 4 | -0.82 | 0.000 | -1.12 | -0.51 | 63% | 0.051 |
|  |  |  |  |  |  |  |  |  | Other geriatric assessment | 1 | -0.47 | 0.000 | -0.67 | -0.27 | 0% | 1.000 |
|  |  |  |  |  |  |  |  |  | Genetically modified models | 2 | -5.82 | 0.000 | -7.12 | -4.53 | 0% | 0.569 |
|  | | | | | | | | | | | | | | | | |
| **Non-heme iron (tissue)** | 4 | 2.82 | 0.000 | 1.47 | 4.17 | 73% | Species | NA | Rat | 4 | 2.82 | 0.000 | 1.47 | 4.17 | 73% | 0.014 |
|  |  |  |  |  |  |  | Frailty assessment | 0.000 | Assumed based on age | 3 | 3.27 | 0.000 | 1.67 | 4.86 | 71% | 0.036 |
|  |  |  |  |  |  |  |  |  | Immobilisation/ sedentary lifestyle | 1 | 1.67 | 0.004 | 0.53 | 2.80 | 0% | 1.000 |
|  | | | | | | | | | | | | | | | | |
| **Transferrin (in serum)** | 7 | -0.34 | 0.255 | -0.94 | 0.25 | 95% | Species | NA | Human | 7 | -0.34 | 0.255 | -0.94 | 0.25 | 95% | 0.000 |
|  |  |  |  |  |  |  | Frailty assessment | 0.806 | Sarcopenia/ muscle atrophy | 1 | -0.07 | 0.736 | -0.51 | 0.36 | 0% | 1.000 |
|  |  |  |  |  |  |  |  |  | Assumed based on age | 2 | -0.88 | 0.050 | -1.76 | -0.00 | 94% | 0.000 |
|  |  |  |  |  |  |  |  |  | Immobilisation/ sedentary lifestyle | 2 | 0.08 | 0.948 | -2.30 | 2.46 | 97% | 0.000 |
|  |  |  |  |  |  |  |  |  | Fried frailty index | 1 | -0.23 | 0.091 | -0.49 | 0.04 | 0% | 0.480 |
