# Supplementary material for: A systematic review and meta‐analysis of studies that have evaluated the role of mitochondrial function and iron metabolism in frailty
Source: Clin Transl Sci. 2021 Jul 9;14(6):2370–8. doi: 10.1111/cts.13101 (PMC8604243; doi:10.1111/cts.13101)
Supplement: Supplementary file 6 — Table S1 [file CTS-14-2370-s001.docx]

| **Database** | **Searched terms** |
| --- | --- |
| **PubMed** | (frail* OR “frailty syndrome” OR “frail elderly” OR sarcopenia) AND (iron OR dmt1 OR ferroportin OR transferrin OR hepcidin OR “circulation iron” OR anaemia OR mitochondria* OR gdf15 OR vimentin OR Idh) |
| **Cochrane library** | (frail* OR “frailty syndrome” OR “frail elderly” OR sarcopenia) AND (iron OR dmt1 OR ferroportin OR transferrin OR hepcidin OR “circulation iron” OR anaemia OR mitochondria* OR gdf15 OR vimentin OR Idh) |
| **Scopus** | (TITLE-ABS-KEY (frail* OR “frailty syndrome” OR “frail elderly” OR sarcopenia)) AND (TITLE-ABS-KEY (iron OR dmt1 OR ferroportin OR transferrin OR hepcidin OR “circulation iron” OR anaemia)) OR (TITLE-ABS-KEY (mitochondria* OR gdf15 OR vimentin OR Idh)) |
| **Ovid Medline** | (sarcopenia.pm. or exp Sarcopenia/ or exp Frail elderly/ or exp Frailty/ or frail*.pm.) AND (mitochondria.mp. or exp Mitochondria/ or exp Growth Differentiation Factor 15/ or GDF15.mp. or FNDC5.mp. or Vimentin.mp. or exp Vimentin/ or LDH.mp. or exp L-Lactate Dehydrogenase/ or exp Anaemia, Iron-Deficiency/ or exp Iron/ or exp Ferritins/ or iron metabolism.mp. or exp Anaemia or DMT1.mp. or Transferrin.mp. or exp Transferrin/ or exp Receptors, Transferrin/ or Ferroportin.mp. or exp Hepcidin/ or exp Hepcidins/ or serum iron.mp.) |
| **BioRXiv** | frail* and iron metabolism  frail* and mitochondrial function |

**Table 1: Online databases and searched terms used to identify studies for this review**
